# Diverse branching forms regulated by a core auxin transport mechanism in plants

**DOI:** 10.1242/dev.201209

**Published:** 2023-03-15

**Authors:** Victoria M. R. Spencer, Lucy Bentall, C. Jill Harrison

**Affiliations:** School of Biological Sciences, University of Bristol, 24 Tyndall Avenue, Bristol, BS8 1TQ, UK

**Keywords:** Branching, Bifurcation, PIN proteins, Auxin transport, *Selaginella*, Plant evo-devo

## Abstract

Diverse branching forms have evolved multiple times across the tree of life to facilitate resource acquisition and exchange with the environment. In the vascular plant group, the ancestral pattern of branching involves dichotomy of a parent shoot apex to form two new daughter apices. The molecular basis of axillary branching in *Arabidopsis* is well understood, but few regulators of dichotomous branching are known. Through analyses of dichotomous branching in the lycophyte, *Selaginella kraussiana*, we identify PIN-mediated auxin transport as an ancestral branch regulator of vascular plants. We show that short-range auxin transport out of the apices promotes dichotomy and that branch dominance is globally coordinated by long-range auxin transport. Uniquely in *Selaginella*, angle meristems initiate at each dichotomy, and these can develop into rhizophores or branching angle shoots. We show that long-range auxin transport and a transitory drop in PIN expression are involved in angle shoot development. We conclude that PIN-mediated auxin transport is an ancestral mechanism for vascular plant branching that was independently recruited into *Selaginella* angle shoot development and seed plant axillary branching during evolution.

## Teaser

**Summary:** This study identified PIN-mediated auxin transport as an ancestral regulator of branching within the vascular plants.

## INTRODUCTION

Branching architectures have evolved many times in different phyla to enable organisms to optimise resource acquisition and exchange with the environment (Harrison, 2017b; Coudert et al., 2019a). In land plants, branching forms originated and diversified independently in the haploid gametophyte and diploid sporophyte stages of the life cycle (Harrison, 2017b). Although gametophyte branching evolved multiple times in the filamentous, thallose or shoot-like forms of bryophytes and ferns, sporophyte shoot branching is thought to have had a single origin in the last common ancestor of vascular plants (Harrison, 2017b) – in the bryophyte sister clade to vascular plants, sporophytes are unbranched with a single stem terminating in reproductive sporangium development. Early land plant fossils such as *Partitatheca* otherwise resemble bryophyte sporophytes but are branched, and early vascular plant fossils such as *Cooksonia* have similar forms but are larger with repeated branching (Harrison, 2017a; Edwards et al., 2021, 2022). This ancestral pattern of branching in vascular plants is known as dichotomous branching, whereby a parent shoot apex splits to form two daughter apices which then grow equally (isotomous branching) or unequally (anisotomous branching) and is shown by living lycophytes and ferns and their ancestors. Branch initiation from leaf axils (axillary branching) was a later independent innovation of seed plants (Harrison and Morris, 2018). The innovation of dichotomous branching in the last common ancestor of vascular plants led to a tenfold increase in plant species numbers and the radiation of diverse plant forms (Harrison and Morris, 2018). Thus, identifying genetic mechanisms enabling the origin and diversification of vascular plant branching patterns is a key goal of evolutionary biologists.

Most of our knowledge of the genetic mechanisms regulating sporophyte branching was generated in flowering plants such as *Arabidopsis*, where branch initiation is intimately linked to leaf initiation at the shoot apex in axillary branching (Domagalska and Leyser, 2011). Short-range PIN-mediated auxin transport away from the axils of initiating leaves generates auxin minima and enables the establishment of axillary meristems (Wang et al., 2014a,b). Axillary meristems can remain dormant for long periods, and branch outgrowth in different parts of the shoot system is globally coordinated by long-range PIN-mediated auxin transport in the stems (Müller and Leyser, 2011). Active shoot apices act as auxin sources, exporting auxin basipetally via the polar auxin transport stream of the stem vasculature (Thimann and Skoog, 1933). This basipetal auxin flow blocks the capacity for auxin export from dormant axillary meristems (Prusinkiewicz et al., 2009). However, if basipetal auxin transport is disrupted, e.g. by excision of the shoot apices, the axillary buds can export auxin, enabling branch outgrowth (Thimann and Skoog, 1933; Prusinkiewicz et al., 2009).

Although these roles for PIN-mediated polar auxin transport in branch initiation and outgrowth are well known in *Arabidopsis*, mechanisms for branching are poorly understood in other plant groups and branching reflects diverse patterns of development (Harrison, 2017a; Coudert et al., 2019a). For example, the filamentous tissues of moss (*Physcomitrium patens*) gametophytes are branched with dominance exerted by the apical cell of each filament (Viaene et al., 2014; Coudert et al., 2019b; Nemec-Venza et al., 2022). PIN-mediated auxin transport out of the apical cells suppresses the development of elongated foraging filaments with strong apical dominance, giving plants a more uniform, circular form (Viaene et al., 2014; Nemec-Venza et al., 2022). In *P. patens* leafy shoots, branches initiate from epidermal cells adjacent to leaves, and diffusive auxin transport from an apical auxin source determines the branching pattern. However, moss leafy shoot branching is analogous to axillary branching in seed plants as their leaves have different origins (Coudert et al., 2015; Thelander et al., 2022). In *P. patens* sporophytes, disruption of PIN-mediated polar auxin transport can induce branching, leading plants to resemble the earliest sporophytic branching forms in the fossil record (Bennett et al., 2014b; Edwards et al., 2014; Harrison and Morris, 2018). Although these data implicate auxin transport in the evolution of diverse branching forms, they were generated using a single species, and mosses are distantly related to *Arabidopsis* and other seed plants (Puttick et al., 2018). Moreover, gene trees show that PIN proteins diversified independently in bryophytes, lycophytes and euphyllophytes (Bennett et al., 2014a), and PINs therefore have unknown ancestral functions in vascular plants.

Lycophytes are a key group to resolve questions about the evolution of sporophyte branching in land plants (Spencer et al., 2021). They originated over 420 million years ago (Morris et al., 2018), and some species exhibit ancestral branching architectures, closely resembling their fossil relatives (Harrison and Morris, 2018). Lycophytes are the sister group to euphyllophytes and are hence ideally placed in the plant tree of life to identify vascular plant homologies (Spencer et al., 2021). Like ancestral vascular plants, lycophytes branch dichotomously (Gola, 2014), and the cellular basis of branching was resolved through clonal analysis in the lycophyte *Selaginella kraussiana*, where anisotomy generates a dominant major branch overtopping a minor sister branch. Anisotomy involves cyclical expansion of the apical stem cell pool, broadening of the shoot apex and the segregation of apical cells to form two daughter apices before branches diverge; major branches initiate from two apical cells and minor branches initiate from a single apical cell (Harrison et al., 2007; Harrison and Langdale, 2010). The overall branching habit of *S. kraussiana* also reflects the development of angle meristems initiating at each anisotomy. A unique evolutionary innovation of Selaginellales, angle meristems typically produce leafless rhizophores that have gravitropic growth and normally start to develop roots when they reach the soil. However, if the shoot tips above an angle meristem are excised, it can instead develop as an angle shoot, contributing to the overall branching habit (Jernstedt et al., 1994; Banks, 2009; Spencer et al., 2021). Surgical and pharmacological experiments in *Selaginella* identified roles for polar auxin transport in plastic development from angle meristems, but a range of species and experimental designs were used (Williams, 1937; Wochok and Sussex, 1975; Jernstedt et al., 1994; Mello et al., 2019), so it is not clear how broadly applicable inferences are. Auxin has been shown to localise in the stem vasculature of *S. wildenowii* (Wochok and Sussex, 1973), there is long-range basipetal transport in *S. kraussiana* explants (Sanders and Langdale, 2013) and auxin transport inhibition leads to shoot apex termination in *S. kraussiana* (Sanders and Langdale, 2013). Based on this knowledge, we chose *S. kraussiana* as a model system to explore the evolution of branching mechanisms in vascular plants.

Here, we report that auxin transport promotes and coordinates dichotomy and the relative growth of branches throughout the *S. kraussiana* shoot system, and that basipetal auxin transport from the shoot apices suppresses angle shoot development. Of four *S. kraussiana* PIN genes, two (*PINR* and *PINS*) are expressed in the shoot tips, and three (*PINR*, *PINS* and *PINT*) in the stem vasculature. The rate of dichotomy is sensitive to pharmacological inhibition of PIN function, and angle shoot development follows a drop in *PINR* and *PINS* expression. We conclude that PIN-mediated auxin transport is an ancestral regulator of vascular plant branching that was co-opted into the evolution of angle shoot development in lycophytes.

## RESULTS

### Dichotomy and rhizophore production proceed consistently during development

**Fig. 1.**
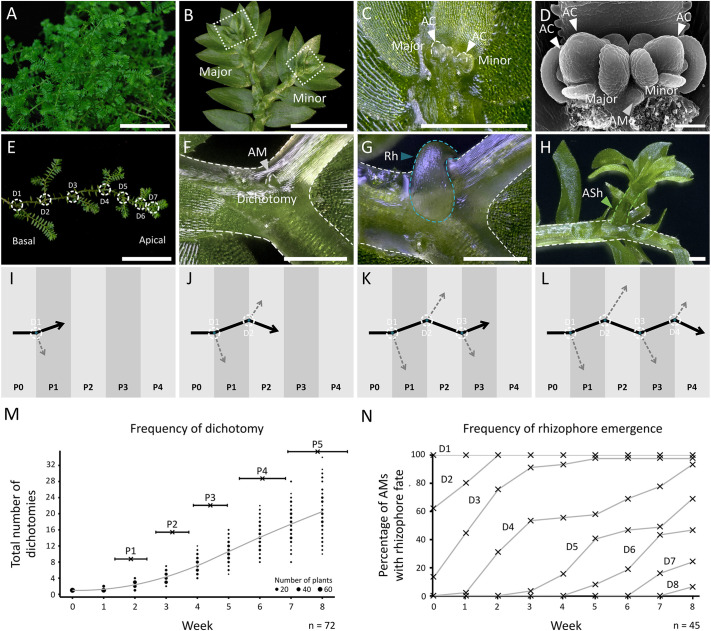
**Branching properties of the lycophyte, *Selaginella kraussiana*.** (A) Photograph of *S. kraussiana*, showing its dichotomising shoot system. (B) Light micrograph showing major and minor branches. Boxes in B indicate apices dissected and magnified in C and D. (C) Light micrograph showing major and minor shoot apices. Asterisks show the position of dissected leaf scars. (D) Scanning electron micrograph showing major and minor shoot apices with apical cells. Asterisks highlight dissected leaf scars. (E) Image showing successive dichotomies (D; white circles) and strong apical dominance in the *S. kraussiana* shoot. (F-H) Light micrographs of a dorsal angle meristem (F), an emerging rhizophore (G) and an emerging angle shoot (H) at a dichotomy. (I-L) Schematic showing that successive dichotomies generate major (black) and minor (grey) branches in *S. kraussiana*. The interval between dichotomies was defined as the dichotomy plastochron (P1-P4), and arrows represent actively growing apices. (M) Graph showing the frequency of dichotomy (P1-P5) in *S. kraussiana* cuttings grown on soil for 8 weeks. The mean plastochron duration is marked (black cross) and the horizontal error bars show standard deviation. Dot size represents the frequency of dichotomy, whilst the fitted line shows a local regression. Data pooled from two experimental replicates (see Fig. S2 for further data). *N*=72. (N) Percentage of angle meristems bearing rhizophores at a given dichotomy. Explants at week 0 had four dichotomies (D1-4) and at the base of each explant (D1) all bore a rhizophore. By week 8, there were rhizophores at the five most basal dichotomies (D1-5). *N*=45. Data pooled from two experimental replicates (see Fig. S2 for further data). AC, apical cells; AM, angle meristem; ASh, angle shoot; Rh, rhizophore. Scale bars: 5 cm (A,E); 5 mm (B); 0.5 mm (C,F-H); 50 µm (D).

*S. kraussiana* has sprawling prostrate shoots (Fig. 1A), which branch anisotomously to produce larger ‘major’ branches and smaller ‘minor’ branches (Fig. 1B-D). Due to the left-right alternation of major and minor branches, a zig-zig like architecture is produced (Fig. 1E). At each dichotomy (Fig. 1E,F) angle meristems subsequently initiate, and these typically produce a rhizophore (Fig. 1G; Fig. S1A). However, angle meristems have plastic identity and, in some cases, can instead produce branching angle shoots (Fig. 1H; Fig. S1B). To provide a baseline for comparison of branching phenotypes following experimental interventions, we first characterised patterns of apical dichotomy and angle shoot production in explants grown on soil (Fig. 1I-N; Fig. S2). Explants with four dichotomies were propagated as cuttings, and the total number of dichotomies from the leading apex was recorded in an 8-week time course (Fig. 1M). The developmental time interval (plastochron) between successive dichotomies along the main axis ranged from <1 to >4 weeks and the mean was 1.6 weeks (Fig. S2A,B). The frequency of rhizophore emergence was determined by calculating the percentage of dichotomies bearing a rhizophore at a given time (Fig. 1E,N; Fig. S2C). At week 0, the most basal angle meristems (D1) had all produced a rhizophore, but this percentage decreased with proximity to the leading apex (Fig. 1E,N). During subsequent weeks, successive angle meristems produced rhizophores such that by week 8, the five most basal dichotomies all bore rhizophores (D15, Fig. 1N). Thus, dichotomy and rhizophore production from angle meristems proceeded in predictable patterns.

### Apical auxin and auxin transport inhibition suppress apical growth and dichotomy

**Fig. 2.**
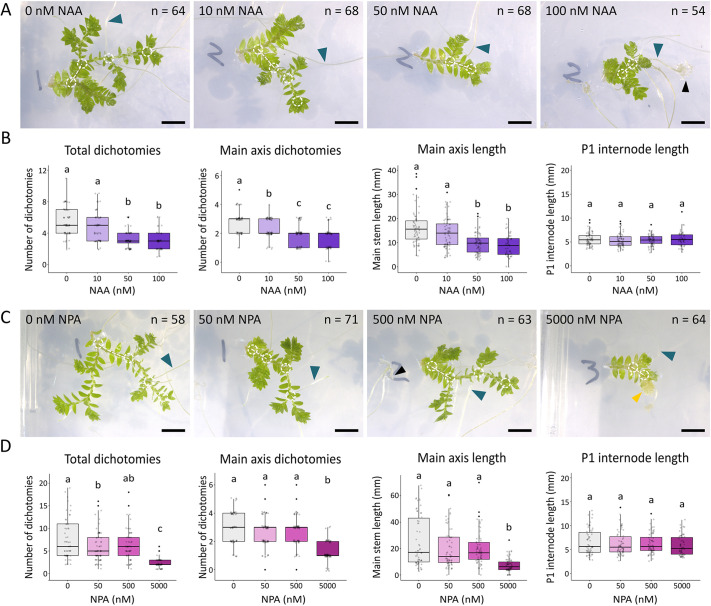
**Short-range auxin transport regulates apical growth and dichotomy.** (A) Images of *S. kraussiana* explants grown for 8 weeks on tissue culture media containing 0 nM, 10 nM, 50 nM and 100 nM NAA. White circles mark main axis dichotomies and white lines show plastochron 1 (P1) internode. Blue arrowhead indicates a rhizophore; black arrowhead indicates a root. (B) Graphs showing that NAA treatment suppressed total and main axis dichotomy and main axis length, but not the internode length of plastochron 1. *N*=64 for 0 nM NAA; 68 for 10 nM NAA; 68 for 50 nM NAA; 54 for 100 nM NAA. One-way ANOVA tests with Tukey multiple comparisons were performed [*F*(3, 233)=38.36, *P*<0.0001; *F*(3, 250)=27.44, *P*<0.0001; *F*(3, 249)=24.45, *P*<0001; *F*(3, 248)=0.72, *P*=0.54]. (C) Images of *S. kraussiana* explants grown for 8 weeks in tissue culture media containing 0 nM, 50 nM, 500 nM and 5 µM NPA. White circles mark main axis dichotomies and lines show plastochron 1 internode. Blue arrowhead indicates a rhizophore; black arrowhead indicates a root; yellow arrowhead indicates a callus. (D) Graphs showing that NPA treatment suppressed total and main axis dichotomy and main axis length, but not the internode length of plastochron 1. *N*=58 for 0 nM NPA; 71 for 50 nM NPA; 63 for 500 nM NPA; 64 for 5000 nM NPA. One-way ANOVA tests with Tukey multiple comparisons were performed [*F*(3, 251)=28.38, *P*<0.0001; *F*(3, 244)=35.39, *P*<0.0001; *F*(3, 245)=19.61, *P*<0.0001; *F*(3, 237)=1.05, *P*=0.37]. In all graphs, boxes represent lower quartile, median and upper quartile, lines represent spread of data and black points show outliers. Data pooled from three experimental replicates (see Fig. S3 for further data). For multiple comparisons (ANOVA), bars bearing different lower case letters are statistically different from one another with a minimum *P*-value of <0.05. Scale bars: 5 mm.

To identify potential roles for auxin and PIN-mediated auxin transport in *S. kraussiana* branching architecture, we first evaluated the effects of exogenously applied auxin (1-napthaleneacetic acid; NAA) and auxin transport inhibitors (N-1-naphthylphthalamic acid; NPA) on explants grown for 8 weeks in axenic culture (Fig. 2; Fig. S3). There were dose-dependent decreases in both the overall number of dichotomies and the number of main axis dichotomies, as well as a dose-dependent decrease in the length of the main axis. No effects of NAA or NPA on the length of individual plastochrons (data for plastochron 1 shown in Fig. 2) or on the number of leaves per plastochron were discernible (Fig. S3). We expected that the length of the main axis would remain constant if dichotomy and apical growth were independently regulated but that the number of dichotomies in a given time period would decrease, or that the length of the main axis would decrease while the number of dichotomies in a given time period would remain constant (Fig. S4). The reduction in both traits shown in our data implies coupled regulation of apical growth and dichotomy by auxin. As explants grown with NPA showed a similar response to explants grown with NAA (Fig. 2C,D; Fig. S3B), and auxin transport out of apical cells is likely required for their function (Sanders and Langdale, 2013), we infer that auxin accumulates in the apical cells of NPA-treated plants and that accumulation suppresses apical growth and dichotomy. Hence, short-range auxin transport out of the apical cells is likely to promote dichotomous branching.

### Long-range auxin transport mediates apical dominance and co-ordinates overall branching architecture

**Fig. 3.**
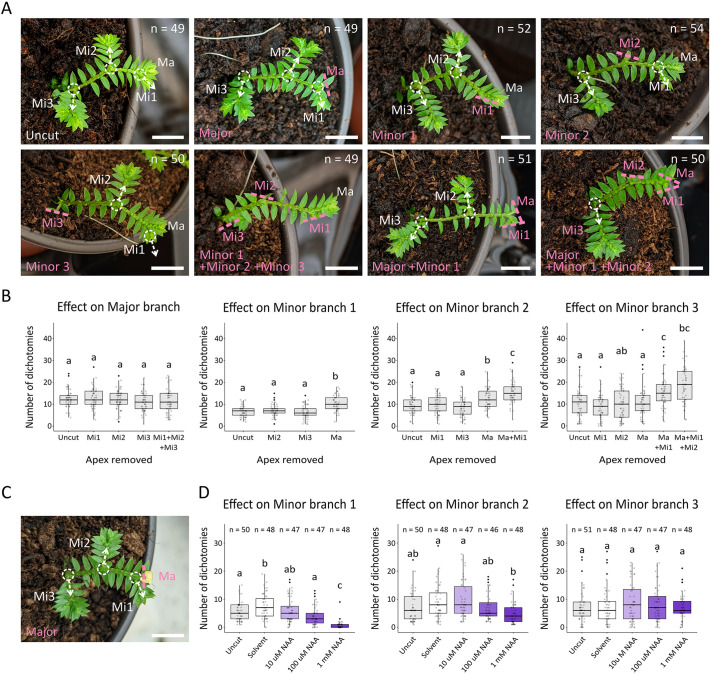
**Long-range auxin transport regulates minor branch dichotomy.** (A) Photographs illustrating experimental design. Explants were uncut, or the major (Ma), first minor branch (Mi1), second minor branch (Mi2) or third minor branch (Mi3) was surgically excised singularly or in combination, and plants were grown for 8 weeks. White dashed circles with arrows show minor branches; pink dashed lines show excision sites. (B) Graphs showing the effect of apex removal on dichotomy. Although minor branch removal had no effect on major branch dichotomy, major branch removal affected dichotomy in minor branches 1 and 2, and jointly with minor branch 1 affected minor branches 2 and 3. *N*=49 for uncut; 49 for major; 52 for minor 1; 54 for minor 2; 50 for minor 3; 49 for minor 1+2+3; 51 for major+minor 1; 50 for major+minor 1+2. One-way ANOVAs with Tukey tests for multiple comparisons were performed [*F*(4, 249)=1.51, *P*=0.2; *F*(3, 198)=18.46, *P*<0.0001; *F*(4, 247)=21.12, *P*<0.0001; *F*(5, 296)=16.38, *P*<0.0001]. (C) Image of an explant showing major branch removal and replacement with lanolin paste. (D) Graphs showing that auxin inhibited dichotomy of minor branch 1 and 2 following excision and replacement of the major branch with lanolin paste containing a solvent control or auxin (10 µM NAA, 100 µM or 1 mM NAA) and 8 weeks of growth. *N*=50 for minor branch 1 uncut; 48 for solvent; 47 for 10 µM NAA; 47 for 100 µM NAA; 48 for 1 mM NAA; 50 for minor branch 2 uncut; 48 for solvent; 47 for 10 µM NAA; 46 for 100 µM NAA; 48 for 1 mM NAA; 51 for minor branch 3 uncut; 48 for solvent; 47 for 10 µM NAA; 47 for 100 µM NAA; 48 for 1 mM NAA. One-way ANOVAs with Tukey tests for multiple comparisons were performed [*F*(4, 235)=24.77, *P*<0.0001; *F*(4, 234)=5.8, *P*=0.00018; *F*(4, 236)=0.66, *P*=0.62]. In all graphs, boxes represent lower quartile, median and upper quartile, lines represent spread of data and points show outliers. Data pooled from three experimental replicates (see Fig. S5 for further data). For multiple comparisons (ANOVA), bars bearing different lower case letters are statistically different from one another with a minimum *P*-value of <0.05. Scale bars: 5 mm.

To identify any long-range effects of auxin and auxin transport on *S. kraussiana* architecture, we implemented a series of surgical and pharmacological experiments on explants grown on soil (Fig. 3; Fig. S5). These explants had three dichotomies, a leading major branch and three minor branches (Ma and Mi1-3, respectively, in Fig. 3A). The major branch and/or the leading minor branch (Mi1), the second side branch (Mi2) or the third side branch (Mi3) was removed (Fig. 3A), and the number of dichotomies in the remaining branches was quantified following 8 weeks of growth (Fig. 3B; Fig. S5A). Although there was no effect of minor branch excision on major branch dichotomy, excision of the major branch tips increased dichotomy in minor branches 1 and 2 but not minor branch 3 (Fig. 3B). However, when minor branch 1 was excised as well as the major branch, the total number of dichotomies in minor branch 3 increased significantly (Fig. 3B). These data suggest that minor branch outgrowth is regulated by long-range basipetal movement of a suppressive signal from the branch tips, that the major branches are the strongest source of the signal, that minor branches are also a source of the signal, and that the signal does not move acropetally. To test the hypothetical identity of this long-range signal as auxin, surgical experiments were combined with pharmacological experiments applying NAA in lanolin paste to excision sites (Fig. 3C). NAA applied at the site of the excised major branch tip suppressed dichotomy of minor branch 1 and minor branch 2 but had a weaker effect on minor branch 3, consistent with an identity of the basipetal signal as auxin (Fig. 3D; Fig. S5B). To determine whether long-range auxin signalling regulates both apical growth and dichotomy in minor branches, the number of main axis dichotomies and length of minor branch 2 were measured. Following major branch decapitation, replacement of the excised apex with 1 mM NAA inhibited minor branch dichotomy and growth (Fig. S6A,B), further supporting our conclusion that apical growth and dichotomy are coupled and regulated by auxin and long-range auxin transport.

### Auxin transport regulates the fate of angle meristems

**Fig. 4.**
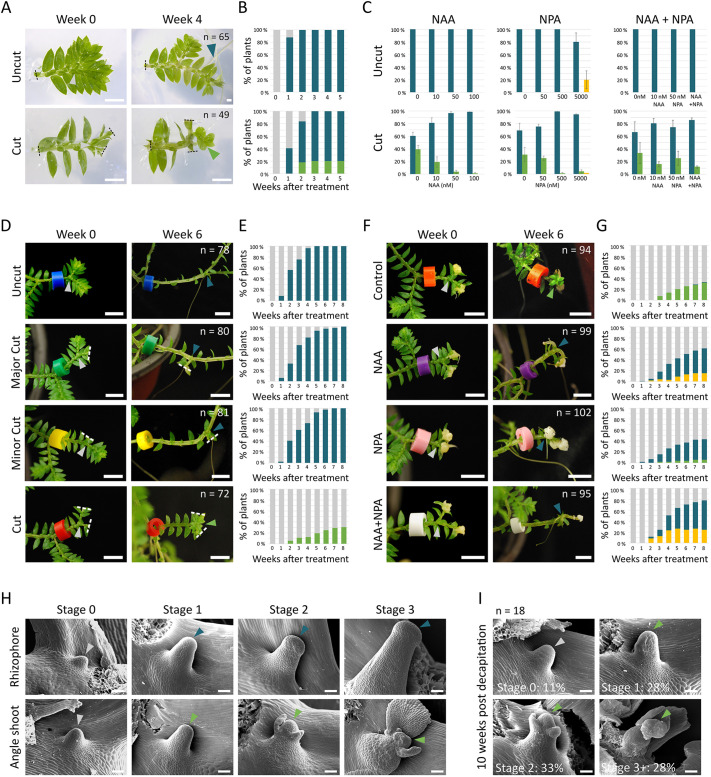
**Apical auxin inhibits angle shoot identity.** (A) Images showing tissue culture grown *S. kraussiana* explants with one dichotomy and apices left intact (uncut) or excised (cut). Uncut explants developed rhizophores (blue arrowhead) by week 2 in a 5-week time course. Cut explants developed rhizophores or angle shoots (green arrowhead). (B) Graphs showing the frequency of rhizophore and angle shoot development from explants shown in A. *N*=65 for uncut; 49 for cut. (C) Explants with one dichotomy were grown on media containing auxin (0 nM, 10 nM, 50 nM and 100 nM NAA), an auxin transport inhibitor (0 nM, 50 nM, 500 nM and 5 µM NPA) or a combination (0 nM, 10 nM NAA, 50 nM NPA and 10 nM NAA+50 nM NPA) and organ identity at the angle meristem was recorded at week 5. In explants with cut apices, NAA and NPA at increasing concentrations progressively reduced angle shoot identity but increased rhizophore initiation frequency. NPA sometimes promoted callus formation. Mean values from three experimental replicates are shown, except for NAA+NPA uncut which had two experimental replicates (see Fig. S7 for further data). Uncut: *N*=78 for 0 nM NAA; 80 for 10 nM NAA; 81 for 50 nM NAA; 77 for 100 nM NAA; 66 for 0 nM NPA; 71 for 50 nM NPA; 74 for 500 nM NPA; 62 for 5 µM NPA; 30 for 0 nM NAA+NPA; 39 for 50 nM NPA; 29 for 10 nM NAA; 40 for NAA+NPA. Cut: *N*=79 for 0 nM NAA; 77 for 10 nM NAA; 79 for 50 nM NAA; 73 for 100 nM NAA; 88 for 0 nM NPA; 84 for 50 nM NPA; 89 for 500 nM NPA; 69 for 5 µM NPA; 43 for 0 nM NAA+NPA; 42 for 50 nM NPA; 37 for 10 nM NAA; 60 for NAA+NPA. Error bars show standard deviation. (D) Explants with four dichotomies were grown on soil and were left uncut, had both apices cut, or had the major or minor apex cut (dashed white lines show excisions). By week 6, rhizophores were produced in all uncut, major cut and minor cut plants. When both apices were excised, plants produced angle shoots instead of rhizophores. Grey arrows indicate angle meristems; blue arrows indicate rhizophores; green arrow indicates a branch. (E) Graphs showing the frequency of rhizophore and angle shoot development from explants shown in D. Data pooled from three experimental replicates (see Fig. S8 for further data). *N*=78 for uncut; 80 for major cut; 81 for minor cut; 72 for cut. (F) Images of plants following apex removal and replacement with lanolin paste mixed with either a solvent control, 1 mM NAA, 500 µM NPA, or 1 mM NAA+500 µM NPA. Angle shoots developed in control plants, but NAA, NPA and NAA+NPA treatments restored rhizophore production or led to production of a callus-like tissue. Grey arrows indicate angle meristems; blue arrows indicate rhizophores; green arrow indicates a branch. (G) Graphs showing the frequency of rhizophore and angle shoot development from explants shown in F. Data pooled from three experimental replicates (see Fig. S8 for further data). *N*=94 for control; 99 for NAA; 102 for NPA; 95 for NAA+NPA. (H) Scanning electron micrographs of angle meristems during rhizophore and angle shoot development. Angle meristems are first domed on the dorsal stem (stage 0), then elongating in rhizophore and angle shoot initiation (stage 1). Following elongation, rhizophores develop a broad and rounded apex, while angle shoots produce leaf primordia (stage 2). Both rhizophores and angle shoots widen at the base and increase in height (stage 3). *N*=13 for rhizophore samples (uncut); 16 for angle shoot samples (2 weeks after cutting). Grey arrowheads indicate angle meristem; blue arrowheads indicate rhizophore apex; green arrowheads indicate angle shoot apex. (I) Scanning electron micrographs showing that angle meristems in explants grown in soil were either undifferentiated or had committed to angle shoot fate 10 weeks after apex decapitation. *N*=18. Grey arrowhead indicates angle meristem; green arrowheads indicate angle shoot apex. Scale bars: 2 mm (A); 5 mm (D,F); 0.05 mm (H,I).

A further component of the overall *Selaginella* branching habit involves plastic shoot development from the angle meristems. However, previously reported results have used diverse species and experimental systems to evaluate the role of long-range cues in this process (Williams, 1937; Wochok and Sussex, 1975; Jernstedt et al., 1994; Mello et al., 2019). To identify any roles for long-range and polar auxin transport in *S. kraussiana* angle shoot production, we first performed surgical and pharmacological experiments using explants grown in tissue culture (Fig. 4A-C; Fig. S7). Explants with 1 dichotomy were grown for 5 weeks and their apices were left intact (uncut) or excised (cut) (Fig. 4A,B). Whereas all angle meristems developed rhizophores after 2 weeks in ‘uncut’ explants, there was a delay in rhizophore production in ‘cut’ explants (Fig. 4B). Furthermore, 20% of angle meristems developed angle shoots in ‘cut’ explants, consistent with a role for long-range signalling in regulating angle shoot identity (Fig. 4B). To identify any roles for auxin and auxin transport in angle shoot identity, explants were grown on media containing a range of concentrations of NAA (0 nM, 10 nM, 50 nM, 100 nM), NPA (0 nM, 50 nM, 500 nM, 5 µM) or NAA and NPA in combination (10 nM NAA+50 nM NPA), and angle meristem fate was recorded at week 5 (Fig. 4C; Fig. S7). Regardless of the concentration of NAA or NPA, ‘uncut’ explants showed no angle shoot production, but with 5 µM NPA, explants developed a callus-like tissue at low frequency (Fig. 4C). In contrast, ‘cut’ explants showed progressive inhibition of angle shoot development and restoration of rhizophore production with increasing concentrations of NAA and NPA (Fig. 4C). Thus, we infer that auxin generated in the shoot apices promotes rhizophore identity but normally represses angle shoot development, and that decapitation releases this repression.

### Long-range apical and basal cues regulate angle meristem identity

To corroborate findings from the tissue culture system and discern any dominance effects of major and minor branch apices on branch emergence from angle meristems, we next implemented surgical and pharmacological experiments in cuttings with four dichotomies grown for 4 weeks on soil (Fig. 4D,E; Fig. S8A). The major axis of each plant was tagged, and either (1) both apices were left intact, ‘uncut’, (2) the major apex was excised, ‘major cut’, (3) the minor apex was excised, ‘minor cut’ or (4) both apices were excised, ‘cut’ (Fig. 4D,E). The activity of angle meristems was subsequently monitored over 8 weeks (Fig. 4D,E). In ‘uncut’ plants, all angle meristems developed rhizophores following 5 weeks of growth, showing a similar pattern of development to the tissue culture system, and removal of either the major or the minor apex resulted in no conspicuous difference to ‘uncut’ plants (Fig. 4B,D,E). As in the tissue culture system, ‘cut’ plants with both the major and minor apices removed developed angle shoots at a low frequency, implicating mobile signals from the shoot tips in angle meristem plasticity and suppression of angle shoot identity. However, unlike the tissue culture system (Fig. 4A-C), no rhizophore emergence followed decapitation in ‘cut’ plants in the whole plant system (Fig. 4B,E).

### The shoot apices are the main source of auxin

To investigate roles for apical auxin and long-range auxin transport in angle shoot development, we combined surgical experiments with a pharmacological approach applying NAA, NPA or a combination of NAA and NPA to excision sites (Fig. 4F,G; Fig. S8B). Following decapitation and 8 weeks of growth, plants with both tips excised and replaced with lanolin paste containing a solvent control produced angle shoots (Fig. 4E,G). As in tissue culture (Fig. 4C), angle shoot development was inhibited in plants with both tips excised and replaced with a lanolin paste containing NAA (Fig. 4F,G), confirming that the apices act as an auxin source regulating angle meristem plasticity and angle shoot identity. However, a callus-like tissue also initiated at a low frequency and rhizophores initiated at a lower frequency than in the tissue culture system (compare Fig. 4C with 4G). To test a role for auxin transport more directly, the auxin transport inhibitor NPA was applied in a lanolin paste to excision sites. Angle shoot production was mostly suppressed by NPA treatment (Fig. 4G), and we considered that phenotype variability may be due to variability in lanolin application efficiency. Applying a lanolin paste with a combination of NAA and NPA did not restore rhizophore development to frequencies higher than the single NAA treatment but increased the frequency of production of the callus-like tissue. Thus, NPA may locally increase auxin levels in the angle meristem to promote callus identity.

### Morphological changes in angle shoot development

To gain further insight into these fate changes, we first characterised morphological changes during rhizophore and angle shoot initiation (Fig. 4H). When the main shoot apices were intact, the angle meristem (stage 0) first elongated (stage 1), before broadening to form a rhizophore apex (stage 2). Subsequent enlargement of the rhizophore apex and positive gravitropism followed (stage 3). However, if the shoot apices were removed, the angle meristem clearly attained a divergent fate, producing leaf primordia at stage 2 (Fig. 4H). This divergent developmental trajectory continued as leaves initiated and the base of the angle shoot expanded (Fig. 4H). In decapitation experiments, further analysis of angle meristem morphology in explants grown on soil showed that all meristems had angle shoot fate 10 weeks following apex removal (Fig. 4I). The differences between the tissue culture system and whole plant system led us to hypothesise that long-range acropetal as well as basipetal cues may regulate rhizophore development. To test this hypothesis, explants with 1-3 dichotomies were grown in tissue culture (Fig. S9A,B) or on soil (Fig. S9C) and the fate of angle meristems was recorded over 5 weeks. In both growth conditions, rhizophore fate was suppressed when explants had two or more dichotomies, suggesting that both apical and basal signals regulate angle meristem fate.

### Three canonical PINs are expressed in *S. kraussiana* shoots

**Fig. 5.**
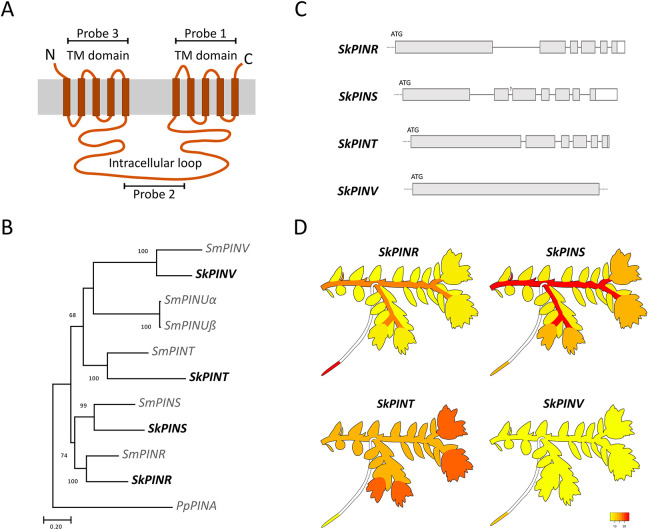
**Three canonical PINs are expressed in *S. kraussiana* shoots.** (A) Diagram showing the canonical structure of lycophyte PINs characterised by a long intracellular loop and N- and C- terminal transmembrane domains and the position of probes used for *in situ* hybridisation. (B) Phylogenetic relationships between *S. moellendorffii* (Sm) and *S. kraussiana* (Sk) PIN genes. Maximum likelihood tree constructed using 226 amino acids of the N-terminal transmembrane domain. Bootstrap values over 50 are shown. A canonical *Physcomitrium patens* PIN gene (*PINA*) was used to root the tree (Bennett et al., 2014a). Genes were named after Bennett et al. (2014a,b) and no orthologues of *S. moellendorffii PINU*α or *PINU*β were detected, but PINU genes may not be expressed (Fig. S10). (C) *S. kraussiana* PIN structures. Grey boxes represent exons, white boxes represent 5′/3′ UTRs and connecting lines represent introns. Dashed lines represent sequences that were not experimentally validated. (D) *In silico* expression analyses using RNA-seq data (Ge et al., 2016) showed tissue-specific expression of *PINS* and *PINT* in the shoot apex, *PINR*, *PINS* and *PINT* in mature stems, and *PINR* and *PINS* in rhizophore apices. *PINV* was not highly expressed in any tissue type. Heat maps represent relative expression levels with undetectable expression represented in yellow and the highest relative expression represented in red.

Using radiolabelled auxin transport assays in cuttings, previously published work has demonstrated that there is NPA-sensitive long-range basipetal auxin transport in the major branch of *S. kraussiana* shoots (Sanders and Langdale, 2013), and such transport is mediated by PIN proteins in *Arabidopsis* (Gälweiler et al., 1998). To identify any potential involvement of PIN-mediated auxin transport in *S. kraussiana* dichotomy, previously identified *S. moellendorffii* PIN peptide sequences were used in reciprocal tBLASTn searches against the *S. kraussiana* genome (Ge et al., 2016), enabling us to identify four *S. kraussiana* PIN genes (Fig. 5A,B). Phylogenetic reconstruction identified *S. kraussiana* PINs as orthologues of *S. moellendorffii PINR*, *PINS*, *PINT* and *PINV* respectively (Bennett et al., 2014a), and they were named accordingly (Fig. 5B). The structure of each PIN (Fig. 5C) was determined by isolating and comparing cDNA sequences to genomic sequences using 3′ RACE, PCR, Sanger sequencing and sequence alignment. Although *S. kraussiana PINR*, *PINS* and *PINT* had multiple introns towards the 3′ end of the gene, no introns were evident in *PINV* (Fig. 5C). To identify PINs with potential roles in branching, we used published RNA-seq data (Ge et al., 2016; (Ferrari et al., 2020) to generate heat maps that showed gross expression in shoot apices, stems, leaves and rhizophores (Fig. 5D; Fig. S10). Although *PINV* showed little to no expression, *PINR*, *PINS* and *PINT* were expressed in multiple shoot tissues. *PINR* and *PINS* were expressed in rhizophores, *PINS* and *PINT* were strongly expressed in shoot apices and *PINR*, *PINS* and *PINT* were strongly expressed in stems (Fig. 5D). Thus, we considered *PINR*, *PINS* and *PINT* candidate regulators of apical growth and dichotomy.

### PINR and PINS are likely regulators of apical growth and dichotomy

**Fig. 6.**
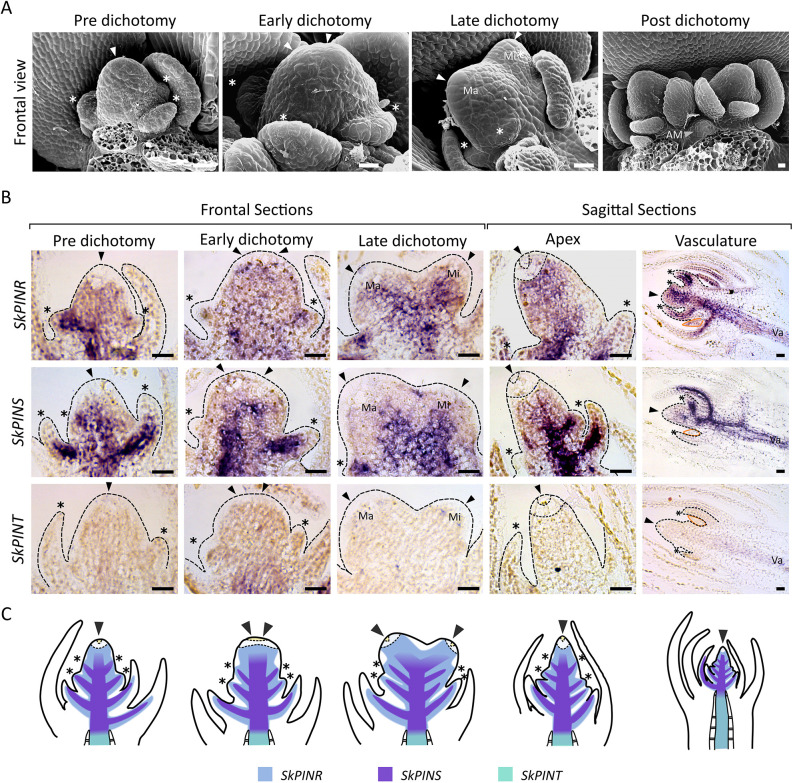
***S. kraussiana PINR* and *PINS* are expressed in shoot apices and stem vasculature.** (A) Scanning electron micrographs of shoot apices undergoing anisotomy. The apical cells duplicate during dichotomy, and then separate to form new major (Ma) and minor (Mi) branch apices (white arrowheads). Angle meristems (AM, grey arrowhead) are evident shortly after dichotomy. Asterisks show initiating leaves. (B) Light micrographs of PIN *in situ* hybridisations during dichotomy. Dashed lines show the edge of the apex, and the major (Ma) and minor (Mi) branches are evident based on their relative size. In sagittal sections, the merophytes and the apical cell are outlined with dashed lines. Arrowheads indicate shoot apices; asterisks indicate initiating leaves; orange outlines indicate developing ligules. Va, vasculature. (C) Schematic summarising the expression patterns shown in B. *PINR* (blue) had broadly apical expression at all stages of dichotomy but was expressed less strongly in the initial cells (yellow) and merophytes (dashed area). *PINS* (purple) had similar expression to *PINR* but expression was more closely associated with the vasculature. *PINT* (green) had no detectable apical expression but was expressed in vascular development. Asterisks indicate developing leaves; black arrowheads indicate shoot apices. Sense controls are shown in Fig. S11. Scale bars: 0.02 mm.

Data shown in Figs 2 and 3 suggest that short-range auxin transport in *S. kraussiana* shoot apices regulates apical cell activity during dichotomy and that long-range auxin transport in the stem vasculature globally co-ordinates plant architecture. Hence, we considered the shoot apices and stem vasculature as likely sites of PIN action. To better resolve spatiotemporal aspects of PIN activity, we used *in situ* hybridisation with probes against the transmembrane domains and intracellular loops (Fig. 5A) to determine gene expression patterns (Fig. 6; Fig. S11). Different stages of the dichotomy cycle in which the initial cells duplicate and then separate to produce two unequal apices were clearly visualised in scanning electron micrographs (Fig. 6A) and *in situ* hybridisation analyses showed strong *PINR* expression across the shoot apex throughout dichotomy, but expression was weaker in the apical cells and merophytes, as confirmed by sagittal sections (Fig. 6B,C; Fig. S11A). *PINS* was also expressed in the shoot apex, but more distally than *PINR* and with a closer association to the developing vasculature. Thus, *PINR* is the strongest candidate regulator of short-range auxin transport during apical growth. Neither *PINS* nor *PINR* showed obvious differential expression in major versus minor apices (Fig. S11B); however, both genes were strongly expressed during leaf initiation and in the leaf vasculature at later stages of leaf development (Fig. 6B,C; Fig. S11B). *PINR*, *PINS* and *PINT* were expressed in the stem vasculature away from the shoot apices (Fig. 6B,C; Fig. S11C), and *PINT* was also expressed in developing ligules (Fig. S11C). We were unable to detect *PINV* expression in any tissue (Fig. S11D). As the vasculature is the likely site of long-range auxin transport, and *PINR* and *PINS* were most highly expressed in this tissue, we concluded that *PINR* and *PINS* together likely co-ordinate long-range auxin transport and overall patterns of shoot architecture.

### PINS and PINT regulate angle meristem plasticity

**Fig. 7.**
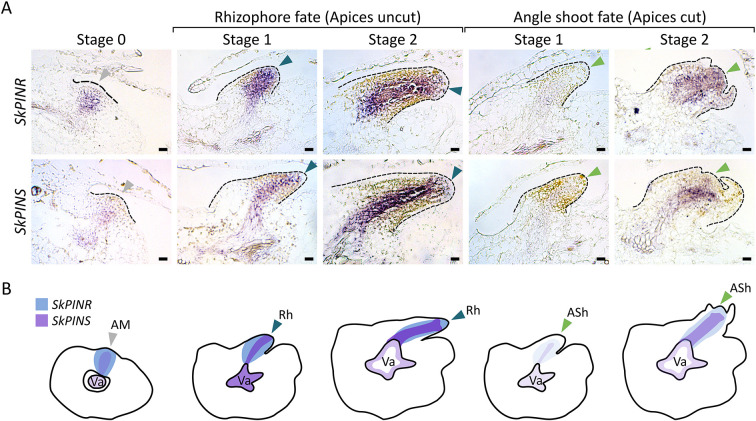
***S. kraussiana PINR* and *PINS* are expressed strongly during rhizophore development.** (A) Light micrographs of RNA *in situ* hybridisation sections of the angle meristem (stage 0) and developing rhizophores and angle shoots (stages 1 and 2). Dashed lines delimit organs; grey arrowheads indicate angle meristem; blue arrowheads indicate rhizophore apex; green arrowheads indicate angle shoot apex. (B) Schematics summarising the expression patterns shown in A. *PINR* (blue) was expressed across the angle meristem (AM, grey arrowhead) and rhizophore apex (Rh, blue arrowheads). *PINS* (purple) had similar expression but was more closely associated with the vasculature (Va). *PINR* and *PINS* were expressed less strongly at stages 1 and 2 of organ emergence than in uncut plants 1 week following surgical decapitation to induce angle shoots (ASh, green arrowheads). Sense controls are shown in Fig. S12. Scale bars: 0.02 mm.

As well as regulating dichotomy, long-range auxin transport through the stem vasculature regulates angle meristem activity. To further investigate the potential involvement of PINs in angle meristem plasticity, we evaluated gene expression patterns at different stages of organ emergence in plants that were fixed with the tips intact or that were fixed 1 week following surgical decapitation (Fig. 7; Fig. S12). We found broad expression of *PINR* in angle meristems before organ emergence (stage 0), but *PINS* was expressed less intensely than *PINR* (Fig. 7; Fig. S12A). *PINR* and *PINS* expression intensified and extended throughout the vasculature as rhizophores initiated (stage 1) and emerged (stage 2) (Fig. 7), while *PINT* and *PINV* expression were undetectable (Fig. S12B). In contrast, *PINR* and *PINS* expression decreased at stage 1 in ‘cut’ plants with angle shoot fate, before expression was restored in the developing angle shoot apex (Fig. 7). We therefore propose that an auxin-regulated decrease in *PINR* and *PINS* expression may be involved in the switch from rhizophore to angle shoot identity. Supporting this notion, *PINR* expression was strong and broader than typical rhizophore expression in plants that had their apices excised and replaced with lanolin paste containing 1 mM NAA (Fig. S12C). The removal of apical auxin following decapitation may therefore cause the drop in PIN expression in stage 1 organs, before restoration of PIN expression generates a basipetal auxin flow in the angle shoot.

## DISCUSSION

### PIN-mediated auxin transport globally co-ordinates dichotomy and angle meristem fate in *S. kraussiana*

**Fig. 8.**
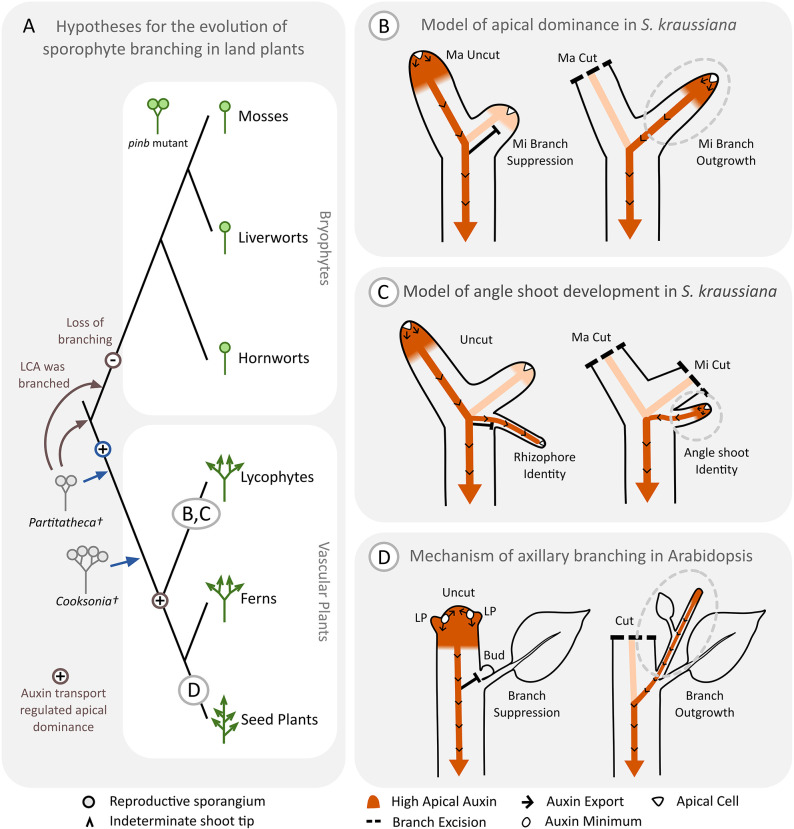
**Hypotheses relating to the regulation of dichotomy and angle shoot identity in *S. kraussiana* and to the evolution of branching mechanisms in land plant sporophytes.** (A) Previous (blue) and current (brown) hypotheses of the origin of dichotomy and the independent evolution of angle shoot branching in lycophytes and axillary branching in seed plants. Eophyte fossils such as *Partitatheca* and early vascular plant fossils such as *Cooksonia* have simple dichotomous branching forms with terminal sporangia (Edwards et al., 2021, 2022), and previous hypotheses (blue arrows and +) proposed that a capacity to dichotomise was acquired in the early steps of vascular plant evolution (Harrison, 2017a; Harrison and Morris, 2018). Together with the finding that disruption of *pinb* function in *Physcomitrium patens* can lead to dichotomy (Bennett et al., 2014b), our finding that PIN-mediated auxin transport is an ancestral branch regulator within vascular plants (brown +) suggests that the common ancestor of land plants may have had a branching form similar to *Partitatheca* (brown arrows), and that dichotomous branching was lost during the evolution of bryophytes (brown −). Phylogeny redrawn from Puttick et al. (2018). (B-D) Models of branch regulation in *S. kraussiana* and *Arabidopsis*. The intensity of the orange shading represents the concentration of auxin, and arrows represent the direction of auxin transport. Black bars represent growth suppression and dashed lines indicates sites of apex excision. (B) In *S. kraussiana*, auxin transport out of the apical cells (white triangles) is likely required to maintain their identity and promote apical growth and dichotomy. The branch apices are auxin sources and major branches produce and export more auxin through the stem vasculature than minor branches (Sanders and Langdale, 2013). Excision of the major branch tips interrupts basipetal auxin transport, enabling auxin from the minor branch tips to access the polar auxin transport stream and releasing minor branch suppression. (C) Lycophytes in the Selaginellales innovated a unique organ system with the capacity to develop as a rhizophore or an angle shoot (Banks, 2009; Spencer et al., 2021), and long-range auxin transport from the shoot apices regulates this plasticity in *S. kraussiana*. If the apices are intact, rhizophores are produced from the angle meristem and likely establish an acropetal auxin transport stream. If the apices are excised, apical auxin and PIN expression in the angle meristem drop, and subsequently an angle shoot is produced. (D) In *Arabidopsis*, auxin export from the leaf axils (white ovals) enables axillary meristem establishment (Wang et al., 2014a,b), and basipetal auxin transport from dominant apices blocks auxin export from axillary buds, suppressing axillary branch outgrowth (Müller and Leyser, 2011). Following surgical excision of dominant apices, axillary buds activate auxin export leading to branch outgrowth (Thimann and Skoog, 1933; Prusinkiewicz et al., 2009). LP, leaf primordium.

We diagrammatically summarise our hypotheses relating to the evolution of branching in Fig. 8A and the mechanisms underlying *S. kraussiana* branching in Fig. 8B-D. Overall, our results lead us to a model whereby short-range PIN-mediated auxin transport out of the apical cells is required to maintain their identity and promote apical growth and dichotomy (Fig. 8B). The rate of dichotomy in major and minor branches is co-ordinated globally, and long-range auxin transport from different parts of the shoot system modulates branch dominance. As supported by radiolabelled auxin transport assays (Sanders and Langdale, 2013), the major branch tips are stronger auxin sources than the minor branch tips, and we propose that they have a greater capacity for auxin export enabling branch dominance (Fig. 8B). Surgical decapitation of the major branch tips enables minor branches to gain dominance (Fig. 8B). *PINR* is likely to provide the short-range auxin transport required to promote dichotomy, and *PINR*, *PINS* and *PINT* are likely to act together to provide long-range auxin transport in the stem vasculature, globally coordinating plant architecture. Auxin export from the shoot tips also modulates the activity of angle meristems and *PINR* and *PINS* are likely to provide the long-range auxin transport required (Fig. 8C). In plants rooted on soil, an interruption to the flow of auxin by decapitation leads to a switch in angle meristem fate and the emergence of angle shoots rather than rhizophores (Figs 4E, 8C), and this switch is associated with a transitory drop in *PINR* and *PINS* expression levels (Fig. 7). Taken together, our results suggest that PIN-mediated auxin transport is a crucial regulator of the overall branching architecture of *S. kraussiana* (Fig. 8).

### Auxin transport out of apical cells affects their activity and identity

Our model of branching regulation is consistent with previous findings that there is long-range auxin transport in the stem in *S. kraussiana* and that NPA treatment leads to apex termination (Sanders and Langdale, 2013). Although previous work noted no link between auxin transport and branching (Sanders and Langdale, 2013), we view this discrepancy as an artefact of different experimental strategies used. Sanders and Langdale (2013) grew explants in shaking liquid culture with NPA, and liquid immersion and the lack of a gravity vector could both affect branching. The regulation of angle shoot production from angle meristems has been studied more extensively than dichotomy, and our results are consistent with previous findings made in other *Selaginella* species that apical auxin suppresses angle shoot identity (Williams, 1937). Topical transport inhibition below the shoot apices in *S. willdenovii* (Wochok and Sussex, 1975) or on the underside of the stem in *S. moellendorffii* (Mello et al., 2019) led to angle shoot production, consistent with the notion that polar auxin transport plays a role. Although basipetal transport in the stem tissues enables rhizophore emergence, transport is acropetal in rhizophores, and this redirection of auxin transport is reflected in patterns of vascular development in many species of *Selaginella* (Matsunaga et al., 2017). Our own experiments attempting to disrupt basipetal transport by applying auxin transport inhibitors around the stem (500 µM NPA in lanolin) yielded no difference in angle meristem identity from controls, we think due to poor tissue penetration (Fig. S13). Our model of angle shoot production from the angle meristems is that, following apical decapitation, a loss in the strength of the auxin transport stream in the stems results in a drop in *PINR* and *PINS* expression in the angle meristems permitting changes in apical cell identity (Fig. 8). We hypothesise that if angle meristem apical cells accumulate auxin they proliferate to form callus or gain rhizophore identity, but if they can export sufficient auxin they produce angle shoots (Fig. 8C), therefore the level of auxin transport out of apical cells may determine their identity.

### PIN-mediated auxin transport was an ancestral regulator of branching in vascular plants

PINs underwent independent duplications in lycophytes and seed plants and there was likely an ancestral *PIN* gene in the vascular plant ancestor (Bennett et al., 2014a,b). In conjunction with findings from *P. patens* that disruption of PIN function can induce dichotomy (Bennett et al., 2014b), our data suggest that PIN-mediated auxin transport is an ancestral regulator of branching within vascular plants (Fig. 8A). As some of the earliest land plant macrofossils comprise dichotomising axes with terminal sporangia but no specialised water conducting cells (e.g. Edwards et al., 2021, 2022), and there is growing evidence of evolutionary loss in the bryophytes (Harris et al., 2020, 2022), we speculate that the single-stemmed state of bryophyte sporophytes was derived from a last common ancestor of land plants manifesting PIN-regulated dichotomy (Fig. 8A). More broadly, many plant groups have evolved multicellular forms with a branching habit and apical dominance (Harrison, 2017a; Coudert et al., 2019a,b). In conjunction with findings presented here, the recent report that *P. patens PINA*, *PINB* and *PINC* regulate apical dominance in moss filament branching (Nemec-Venza et al., 2022) suggests PIN-mediated auxin transport as a core mechanism for branching within land plants. We propose that heterotopic changes in PIN expression or localisation were key drivers of the evolution of diverse branching forms.

## MATERIALS AND METHODS

### Plant growth conditions

*S. kraussiana* (Kunze) A. Braun plants were grown in indirect light at 5-20 µmol m^−2^ s^−1^, 21°C, 70% humidity in long-day conditions (16 h light, 8 h dark). Plants were propagated by transferring apical shoots with at least three major axis dichotomies to soil (Levington Advance Pot and Bedding Compost) and grown in trays sealed in bags to increase humidity. Alternatively, explants were grown in axenic tissue culture with ¼ Gamborg B5 medium (Duchefa Biochemie; G0209.0050), 0.8% Agar (Sigma; A4675-1KG), pH 5.8 and 1% sucrose (Alfa Aesar; A15583), in the same light conditions. To sterilise tissue, *S. kraussiana* explants with ∼2 dichotomies on the major axis were harvested and rinsed with dH_2_O for 15 min. Explants were then incubated in 20% sodium hypochlorite solution (Thermo Fisher Scientific; S/5040/PB17) for 10 min, during which the falcon tube was inverted six times every minute. Tissue was then rinsed four times in sterile dH_2_O before transfer to media. Sterile tissue was propagated in Magenta boxes and hormone experiments were performed in deep Petri dishes of varying sizes.

### Pharmacological experiments

Sterile explants with 1 dichotomy were transferred to deep Petri dishes containing ¼ strength Gamborg B5 media and 0.8% Agar, at pH 5.8 with 1% sucrose (unless stated otherwise). Hormone treatments were performed at concentrations described in the results section. In all cases, solvents were standardised to 0.7% ethanol and 0.02% DMSO. Plants were grown for 8 weeks and photographed for phenotypic analysis. Angle meristem fate was observed once every week to check for the presence or absence of angle shoots and rhizophores over a period of 5 weeks. Samples were excluded if individuals became contaminated or apices were not completely removed.

### Surgical experiments

To investigate dichotomy, explants with three dichotomies of the major axis were transferred to pots and grown in bags. Surgical interventions were made at week 0 and at week 8 plants were scanned using a HP Scanjet G2710 and the number of dichotomies of each minor branch recorded. Using the same set up, surgical experiments were combined with pharmacological treatments as described in the results section. Solvents were standardised to 7% ethanol and 0.2% DMSO in all experiments. To investigate angle meristem activity, explants with four dichotomies of the major axis were transferred to pots as above and grown for four weeks. The most distal shoot tips were decapitated and branches beneath the tips were marked with coloured beads. Plants were either left to grow (‘uncut’) or had the shoot tips excised (‘cut’). Lanolin hormone paste was applied with a syringe to excision points at concentrations stipulated in the results section. Angle meristem activity was observed weekly and the presence of an angle shoot or rhizophore recorded. Treatments were randomly allocated to each plant within a pot. Samples were excluded if individuals became contaminated or lanolin paste was dislodged. Branches were not measured if partially obscured.

### Scanning electron microscopy

Shoot apices and stem sections with a dichotomy were fixed in 2.5% glutaraldehyde (Agar Scientific, R1010) + 0.1 M cacodylate buffer overnight. Tissue was washed three times in dH_2_0, before dehydration in 30% ethanol (15 min), 50% ethanol (15 min), 70% ethanol (10 min), 90% ethanol (10 min), 100% ethanol with molecular sieve (10 min×3). Tissue was critical point dried (CPD300, Leica) before mounting and sputter coating (K757X, Emitech) for 1 min. Samples were imaged at 5-10 kV with a Zeiss Evo 15 ESEM.

### PIN gene identification in *S. kraussiana*

*S. moellendorffii* PIN peptides were used in tBLASTn searches against the *S. kraussiana* genome downloaded from Ge et al. (2016). An e-value cut off of 0.05 was used. The *S. kraussiana* sequence boundaries returned did not fall within annotated regions of the genome, so contigs to which the *S. moellendorffii* gene aligned were downloaded. Each contig was aligned to *S. moellendorffii* PIN genes to find regions of homology likely to encode *S. kraussiana* PIN orthologues. Each putative *S. kraussiana* PIN orthologue was translated and aligned to *S. moellendorffii* orthologues using Clustal X (Thompson et al., 1997). Maximum likelihood (ML) trees were constructed in MEGA X (Kumar et al., 2018) using 226 conserved amino acids in exon 1 with the JTT Model of amino acid substitution (Jones et al., 1992), and including the equivalent sequence from *P. patens* PIN (*PINA*) as an outgroup. The phylogenetic trees generated were used to name *S. kraussiana* PIN genes relative to their closest *S. moellendorffii* orthologue (Bennett et al., 2014a,b). Four PIN genes (*PINR*, *PINS*, *PINT* and *PINV*) were identified.

### RNA-seq gene expression quantification

PIN gene sequences were used in BLAST searches against the *S. kraussiana* genome assembly (Ge et al., 2016), to produce contig coordinates for each gene and create a simplified annotation format (SAF) file. Using these newly created SAF files and bam files kindly provided by the authors of Ge et al. (2016) for each tissue type (stem, leaves, root, shoot apex), the number of reads aligning to specific regions was determined using Featurecounts (Liao et al., 2014). Multimapping was allowed as total counts from multimapping did not differ dramatically from single mapping counts, but did allow quantification of *PINV*, as this gene has three identical sequences on three different contigs. Multimapping counts were used for all genes and normalised using FPKM. Heat map colours were generated with these values using http://www.heatmapper.ca/expression/ and overlayed onto *S. kraussiana* diagrams in Inkscape.

### RNA extraction

A modified Trizol prep was used to extract RNA (Chomczynski and Sacchi, 1987). We mixed 500 µl of Solution D [4 M guanidine isothiocyanate, 25 mM sodium citrate (pH 7.0), 0.5% sarkosyl], 500 µl phenolchloroform-isoamyl alcohol mixture (Sigma-Aldrich; 77618) and 50 µl 2 M sodium acetate (pH 4) and added to 30 mg of frozen ground, *S. kraussiana* shoot tips. The solution was vortexed for 10 s and the supernatant transferred to a new tube. After 5 min at room temperature, 200 µl chloroform:isoamyl alcohol 24:1 (Serva; 39554.02) was added and incubated for 5 min. The samples were centrifuged at 12,000 ***g*** for 15 min at 4°C. Then 500 µl of the top aqueous layer was added to 500 µl isopropanol. After 10 min incubation at room temperature, solutions were mixed by inverting, then centrifuged at 12,000 ***g*** for 10 min at 4°C. The white RNA pellet was washed in 100% ethanol, centrifuged again at 7500 ***g*** for 4 min at 4°C, air dried, then resuspended in 30 µl DEPC H_2_0. RNA quality was checked by gel electrophoresis and concentrations were determined using a NanoPhotometer^®^ N60 (Implen).

### Primers

A list of primers for PCR and cloning is included in Table S1.

### cDNA synthesis

We incubated 1 µg of *S. kraussiana* RNA with DNAse I (Thermo Fisher Scientific; EN0521) and 1× reaction buffer in a total volume of 10 µl for 30 min at 37°C. Then 1 µl of 50 mM EDTA was added and the solution incubated at 65°C for 10 min. This whole reaction was added to Primer Mix (Qiagen; 1030542), 0.5 mM dNTPs, 1× first strand buffer, 0.01 M DTT, RiboLock RNAse Inhibitor (Thermo Fisher Scientific; E0038) and Superscript® II Reverse Transcriptase (Invitrogen; 100004925) to a final volume of 20 µl. The reaction was incubated at 42°C for 50 min and then 70°C for 15 min. A no-RT control sample was also produced for every round of cDNA synthesis to check for contamination.

### PIN CDS cloning

Primers to amplify *S. kraussiana* PIN genes were designed by aligning *S. moellendorffii* CDS to the homologous *S. kraussiana* genomic sequence. Primers were located at regions of high sequence similarity and as close as possible to the predicted ATG and stop codon of the transcript. All primers were checked for low self-dimerisation and heterodimerisation ΔG values using IDT OligoAnalyser software (https://eu.idtdna.com/pages/tools/oligoanalyzer?returnurl=%2Fcalc%2Fanalyzer). PCR was performed with *S. kraussiana* cDNA and Q5^®^ HF DNA Polymerase (New England Biolabs; M0491L). PCR products were separated by gel electrophoresis and extracted, incubated with Taq Polymerase for 30 min at 68°C for A-tail addition, and ligated into pGEM^®^-T Easy Vector (Promega; A137A) overnight at 16°C according to the manufacturer's protocol. After transformation into TOP10 *Escherichia coli* by electroporation, positive colonies were screened by digest or culture PCR, and then sequenced by Eurofins Genomics TubeSeq Service. Sequence results were aligned to the *S. kraussiana* genome, to identify exon and intron positions. The annotated gene sequences are included in Table S2.

### 3′ RACE PCR

To identify the 3′ untranslated region (UTR) of *S. kraussiana* PINs, 3′ RACE PCR was performed. cDNA was synthesised as described above, but using the Q_T_ primer (Frohman, 1990; Frohman and Brook, 1994). The first round of PCR used a forward gene specific primer (GSP1) within a known exon in the middle of the transcript, and the Q_O_ primer (Frohman, 1990). The second round of PCR used the PCR product from the first round, a forward gene specific primer (GSP2) nested further downstream of GSP1, and the Q_I_ primer (Frohman, 1990). Q5 HF DNA Polymerase was used for all reactions according to the manufacturer's guidelines. For the 1st PCR, a 2 min extension with 58°C annealing temperature and 25 cycles were used. For the 2nd PCR, 35 cycles were used. The PCR product from the 2nd PCR was visualised by gel electrophoresis and extracted, incubated with Taq polymerase for 30 min at 68°C, and ligated into pGEM-T Easy overnight at 16°C according to the manufacturer's protocol. After transformation into TOP10 *E. coli* by electroporation, positive colonies were screened by digest or culture PCR, and then sequenced by Eurofins Genomics TubeSeq Service. Sequence results were aligned to the *S. kraussiana* genome to identify the 3′ UTR of each gene.

### RNA probe synthesis

pGEM-T-Easy DNA vectors containing ∼300 bp of each *S. kraussiana* PIN gene in both orientations were amplified using a midiprep kit (Qiagen; 12143). Plasmids were digested with SpeI (New England Biolabs; R3133S) and purified by phenol/chloroform extraction. RNA transcription in sense and antisense orientations was performed with the T7 RNA polymerase (New England Biolabs; M0251S) using digoxigenin11-UTP (Roche; 11209256910) to label the probes. After DNAse treatment, RNA was precipitated with 7.5 M NH_4_Ac and cold ethanol before probe hydrolysis (time depended on length of template). Probe fragments were then precipitated with 3 M NaOAc, 10% HAc and ethanol, and re-dissolved in a 50:50 mix of DEPC-treated H_2_O and formamide. To verify DIG labelling efficiency, probe dilutions were spotted onto a Bright Star Plus Nylon membrane (Invitrogen; AM10102), washed for 5 min in buffer 1 (100 mM Tris-HCl, 150 mM NaCl), blocked for 30 min with buffer 2 [0.5% (w/v) blocking reagent (Roche, 11096176001) in buffer 1], washed three times in buffer 1 for 5 min, and incubated for 30 min in 1:5000 anti-digoxigenin-AP Fab Fragment antibodies (Roche; 11093274910). After washing the membrane twice in buffer 1 for 15 min, the signal was developed using an NBT/BCIP solution (pH 9.5) (Sigma-Aldrich, B5655).

### Tissue fixation for *in situ* hybridisation

Shoot apices and segments of stem with a dichotomy were dissected and fixed in 4% paraformaldehyde (Sigma-Aldrich, 441244) + 4% DMSO overnight at 4°C. Tissue was passed through a 4°C ethanol series (30%, 40%, 50%, 60%, 70%, for 1 h each) before transfer to a Tissue-Tek VIP (Sakura). Tissue was processed as follows: 70% ethanol (1 h), 80% ethanol (1 h), 90% ethanol (1 h), 95% ethanol (1 h), 100% ethanol (1 h), 100% ethanol (1 h), 100% ethanol (1.5 h), 100% Histoclear (National Diagnostics; A2-0101) (1 h), 100% Histoclear (1 h), 100% Histoclear (1.5 h). All of these steps were performed at 35°C with a slow mix. Tissue was transferred to fresh wax for 1.5 h, 2 h, 2.5 h, 2.5 h, all at 60°C. Samples in Fig. S12C were prepared using a Leica ASP300 tissue processor following the same protocol, but the 80% ethanol step was omitted and ROTI^®^ Histol (Roth) used instead of Histoclear. Tissue was embedded in moulds and stored at 4°C until use. Then 8 µm sections were prepared using a Leica RM2245 microtome and left overnight on a 42°C hotplate before use.

### *In situ* hybridisation

Sections were pre-treated as follows: Histoclear (10 min), Histoclear (10 min), 100% ethanol (1 min), 100% ethanol (30 s), 95% ethanol (30 s), 85% ethanol+0.85% saline (30 s), 50% ethanol+0.85% saline (30 s), 30% ethanol+0.85% saline (30 s), 0.85% saline (2 min), phosphate buffered saline (PBS) (2 min), 0.125 mg ml^−1^ Pronase (37°C, 10 min) (Sigma-Aldrich; P2308), 0.2% glycine (2 min) (ChemCruz; sc-29096), PBS (2 min), 4% paraformaldehyde in PBS (10 min), PBS (2 min), PBS (2 min), acetic anhydride (Sigma-Aldrich; 320102) in 0.1 M triethanolamine (pH 8) (10 min) (Sigma-Aldrich; T58300), PBS (2 min), 0.85% saline (2 min), 30% ethanol+0.85% saline (30 s), 50% ethanol+0.85% saline (30 s), 85% ethanol+0.85% saline (30 s), 95% ethanol (30 s), 100% ethanol (30 s), fresh 100% ethanol (30 s). Slides were left to dry for 1-2 h.

Probes were mixed in a 1:4 ratio with hybridisation buffer [300 mM NaCl, 10 mM Tris-HCl (pH 6.8), 10 mM NaPO_4_ buffer, 5 mM EDTA, 50% deionised formamide, 1 mg ml^−1^ tRNA, 1×Denhardt's solution (Sigma-Aldrich; D2532), 10% dextran sulphate (Sigma-Aldrich; D8906)] and applied to slides. Hybridisations were incubated at 50°C overnight, before washing for 1 h 30 min at 50°C in wash solution (15 mM NaCl, 1.5 mM Na_3_C_6_H_5_O_7_). Following two incubations in NTE solution [0.5 M NaCl, 10 mM Tris-HCl (pH 7.5), 1 mM EDTA] for 5 min at 37°C, slides were transferred to NTE+20 µg/ml RNAse A for 30 min at 37°C. Slides were then washed in NTE×3 (5 min each, 37°C), wash solution (1 h, 50°C), and PBS (5 min).

Anti-DIG antibodies were applied and signal developed as follows: 5 min in buffer 1 (100 mM Tris-HCl, 150 mM NaCl), 30 min in buffer 2 [0.5% (w/v) blocking reagent in buffer 1], 30 min in buffer 3 (1% bovine serum albumin, 0.3% Triton X-100 in buffer 1), 1.5 h in buffer 4 (anti-digoxigenin-AP 1:3000 in buffer 3), 4×20 min in buffer 3, 5 min in buffer 1, 5 min in buffer 5 [100 mM Tris (pH 9.5), 100 mM NaCl, 50 mM MgCl_2_], followed by 12 h+ in buffer 6 (NBT/BCIP solution) until the signal develops. The enzymatic reaction was then stopped by washing slides for 30 s each in distilled H_2_O, 70% ethanol, 95% ethanol, 100% ethanol, 95% ethanol, 70% ethanol, and distilled H_2_O. Sections were mounted in Entellan mounting medium (Merck; 1.07961.0500) with coverslips before imaging. Both transmembrane domain probes and intracellular loop probes showed the same expression patterns for each gene.

### Image acquisition

Images of living *S. kraussiana* were taken using a Keyence Digital microscope (VHX-1000E) with a RZ×50 or RX×20-x 200 objective lens, a D80 Nikon camera with a EX Sigma 50 mm 1:2.8 DG MACRO lens, or a Google Pixel 6 phone camera. Images of *in situ* sections were taken using a Leica DM2000 LED microscope with ×20 and ×40 objective lenses and a Leica MC120 HD camera attachment.

### Data analysis

All data were collected and bar graphs produced in Microsoft Excel (2020). Boxplots were produced and one way ANOVA tests with multiple comparisons were performed in R studio (http://www.rstudio.com/). All figures and diagrams were produced using Inkscape (https://inkscape.org/).

## Supplementary Material

10.1242/develop.201209_sup1 Supplementary information
