# Supplementary material for: Diverse branching forms regulated by a core auxin transport mechanism in plants
Source: Development. 2023 Mar 15;150(6):dev201209. doi: 10.1242/dev.201209 (PMC10108033; doi:10.1242/dev.201209)
Supplement: Supplementary information [file develop-150-201209-s1.pdf]

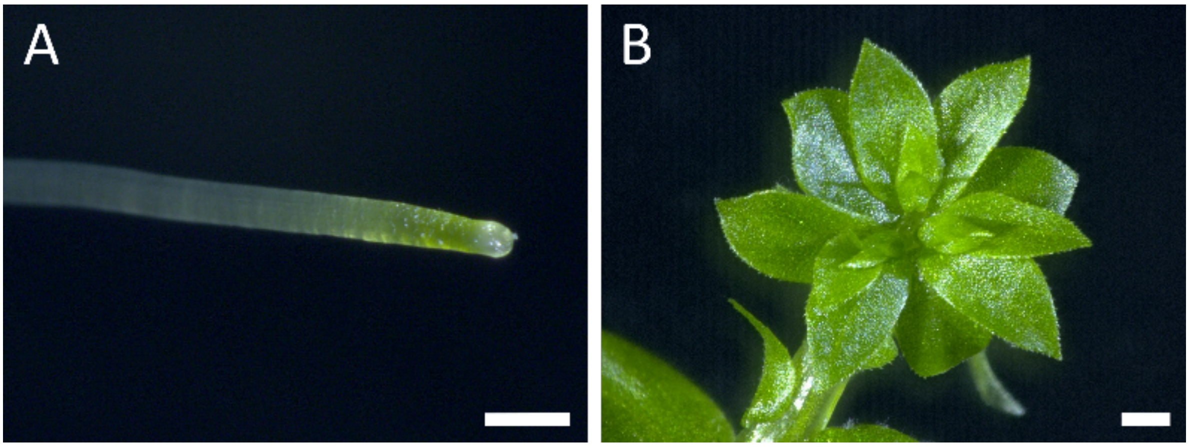

Fig. S1 related to Figure 1. Later stages of rhizophore and angle shoot development (**A**) Light micrograph of the rhizophore, which displays positive gravitropism but lacks root hairs and a root cap. (**B**) Light micrograph of an emerging angle shoot at a shoot dichotomy. Unlike the mature shoot, the angle shoot first displays spiral phyllotaxy before producing leaves in opposite pairs. Scale bar = 0.5 mm.

Development • Supplementary information

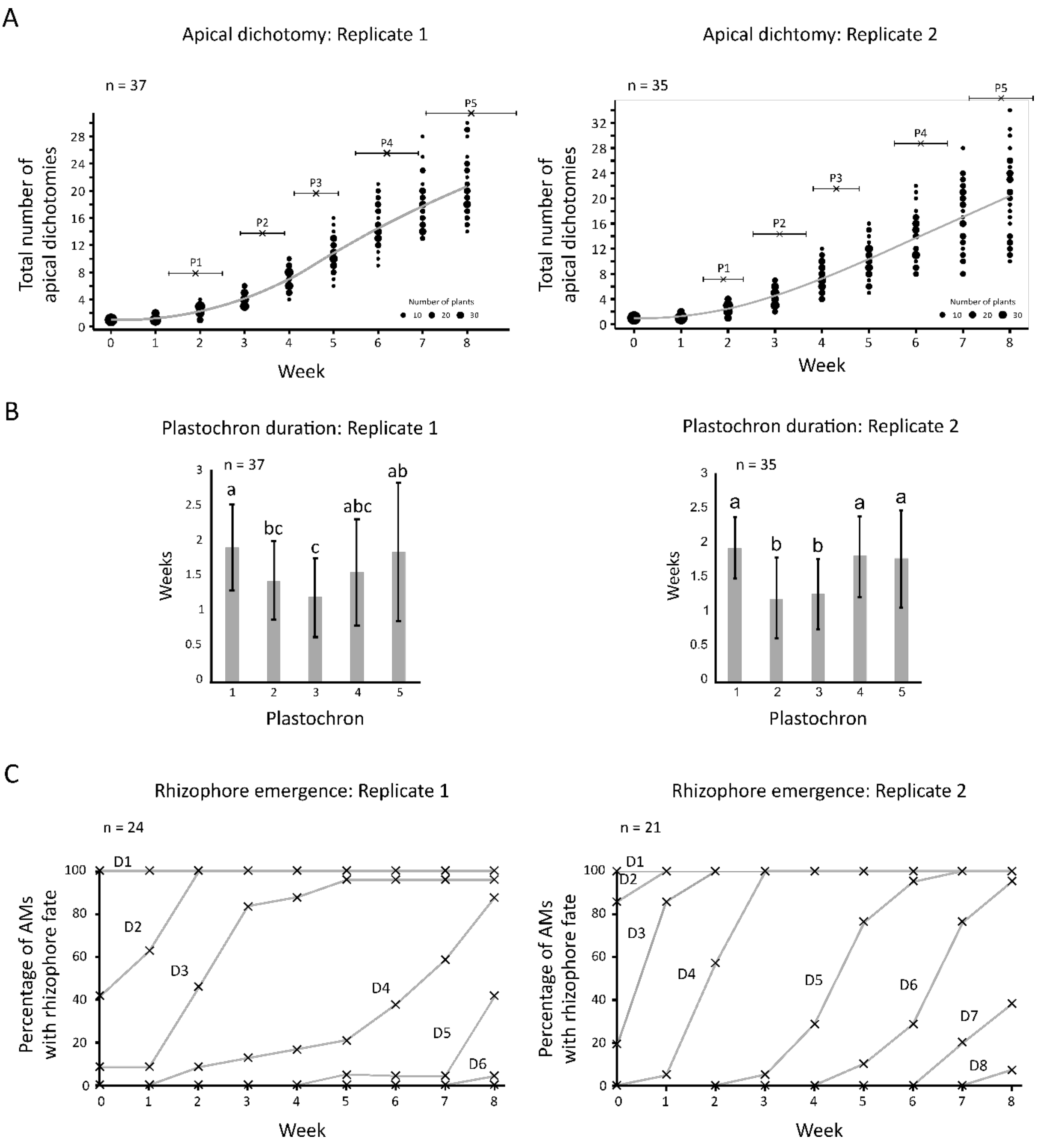

Fig. S2 related to Figure 1. Dichotomous branching and rhizophore emergence time course data from two experimental replicates. (**A**) Graphs showing the total number of dichotomies in an explant following transfer to soil. (**B**) Bar graphs showing the mean duration of plastochrons 1-5. P2 and P3 proceeded more rapidly than P1, P4 and P5. One-way ANOVAs with Kruskal-Wallis tests for multiple comparisons were performed ($\chi^2(4) = 22.87$, $p = 0.00014$; $\chi^2(4) = 42.07$, $p < 0.0001$). Error bars show standard deviation. (**C**) Graphs showing the percentage of angle meristems (AMs) that have produced rhizophores at a given dichotomy (D) during an eight-week time course.

Development • Supplementary information

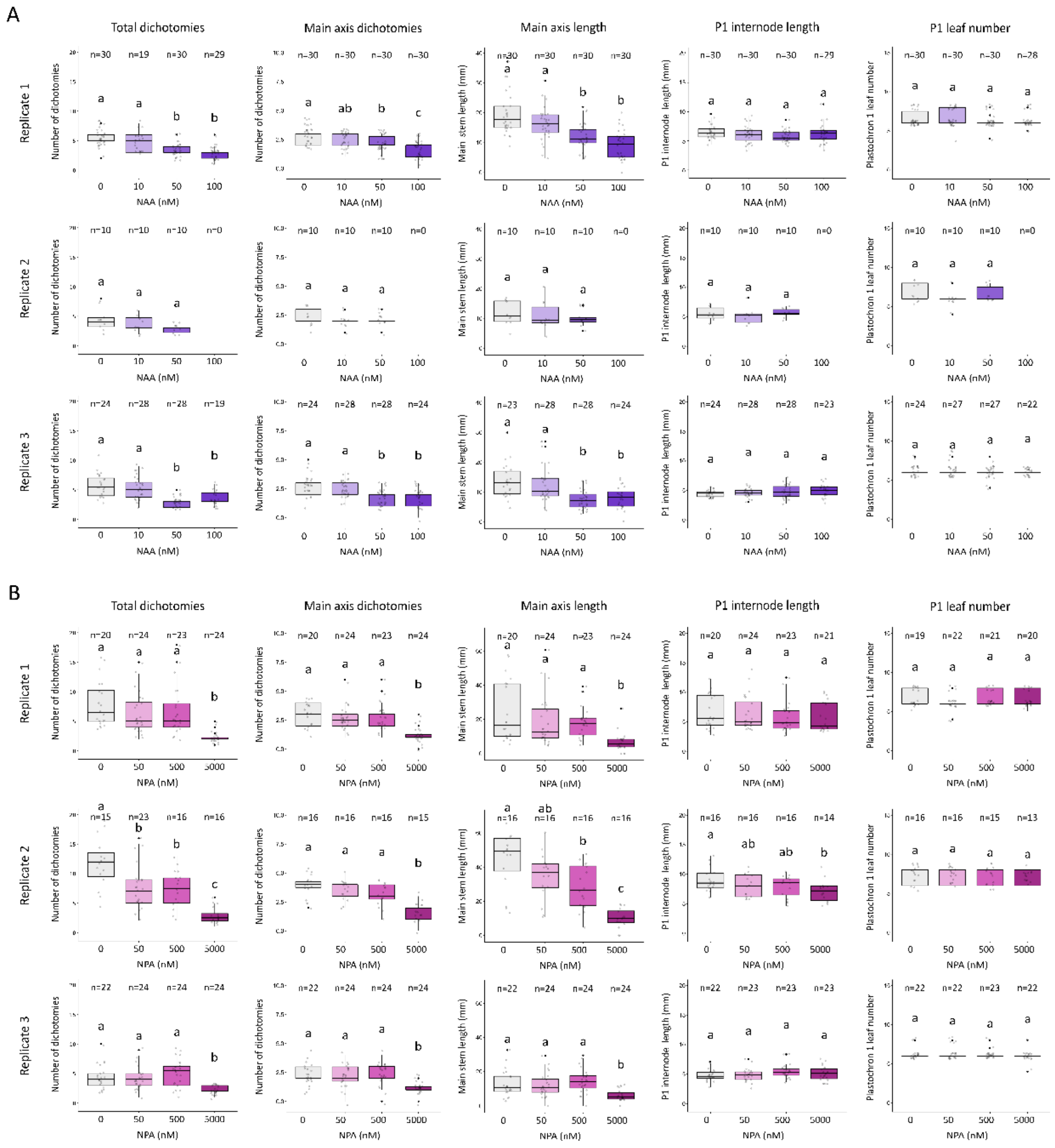

Fig. S3 related to Figure 2. Auxin and auxin transport inhibitor application suppress apical growth and dichotomy. (**A**) Graphs representing data from three experimental replicates of pharmacological experiments using explants with one dichotomy and grown in tissue culture for eight weeks with 0 nM, 10 nM, 50 nM or 100 nM NAA. (**B**) Graphs representing data from three experimental replicates of pharmacological experiments using explants with one dichotomy and grown in tissue culture for eight weeks with 0 nM, 50 nM, 500 nM or 5 μM NPA. In all graphs, boxes represent lower quartile, median and upper quartile. Lines represent spread of data and black points show outliers. One-way ANOVA tests with Tukey or Kruskal Wallis multiple comparisons were performed. (NAA Rep 1: $F(3,104) = 26.21$, $p < 0.0001$; $F(3,116) = 18.9$, $p < 0.0001$; $F(3,116) = 20.44$, $p < 0.0001$; $F(3,115) = 1.33$, $p = 0.27$; $\chi^2(3) = 2.01$, $p = 0.57$. NAA Rep 2: $F(2,27) = 2.94$, $p = 0.07$; $F(2,27) = 0.36$, $p = 0.7$; $F(2,27) = 0.58$, $p = 0.57$; $F(2,27) = 0.66$, $p = 0.53$; $\chi^2(2) = 2.15$, $p = 0.34$. NAA Rep 3: $F(3,95) = 18.2$, $p < 0.0001$; $F(3,100) = 12.26$, $p < 0.0001$; $F(3,99) = 10.79$, $p<0.0001$; $F(3,99) = 1.37$, $p = 0.26$; $\chi^2(3) = 6.13$, $p = 0.1$. NPA Rep 1: $F(3,87) = 12.22$, $p < 0.0001$; $F(3,87) = 16.6$, $p < 0.0001$; $F(3,87) = 7.07$, $p = 0.00026$; $F(3,84) = 0.59$, $p = 0.62$; $\chi^2(3) = 3.05$, $p = 0.38$. NPA Rep 2: $F(3,66) = 17.94$, $p < 0.0001$; $F(3,59) = 17.44$, $p<0.0001$; $F(3,60) = 19.94$, $p < 0.0001$; $F(3,58) = 2.45$, $p = 0.072$; $\chi^2(3) = 0.42$, $p = 0.94$. NPA Rep 3: $F(3,90) = 11.86$, $p < 0.0001$; $F(3,90) = 9.79$, $p < 0.0001$; $F(3,90) = 7.48$, $p = 0.00016$; $F(3,87) = 1.89$, $p = 0.14$; $\chi^2(3) = 4.3$, $p = 0.23$).

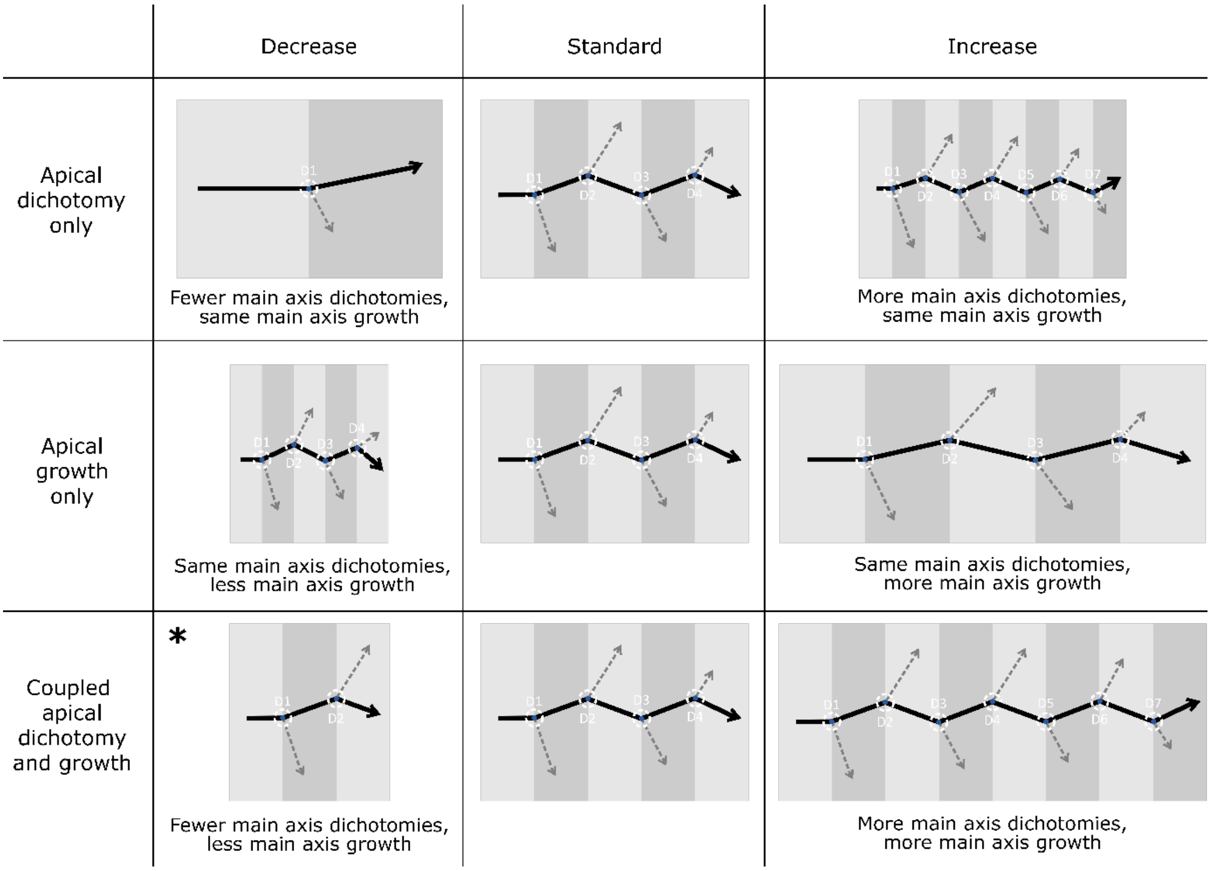

Fig. S4 related to Figure 2. Growth phenotypes expected during a specified time frame if dichotomy and growth are regulated separately or are coupled. Alternating pale and dark grey boxes show the internode lengths between apical dichotomies. White dashed circles surround the dichotomy (D) and blue dots represent the angle meristem. The main stem axis is represented by a solid black line, whilst side branches are represented by dashed grey lines. Arrowheads represent growing apices. Asterisk indicates the phenotype observed after exogenous NAA and NPA application in tissue culture.

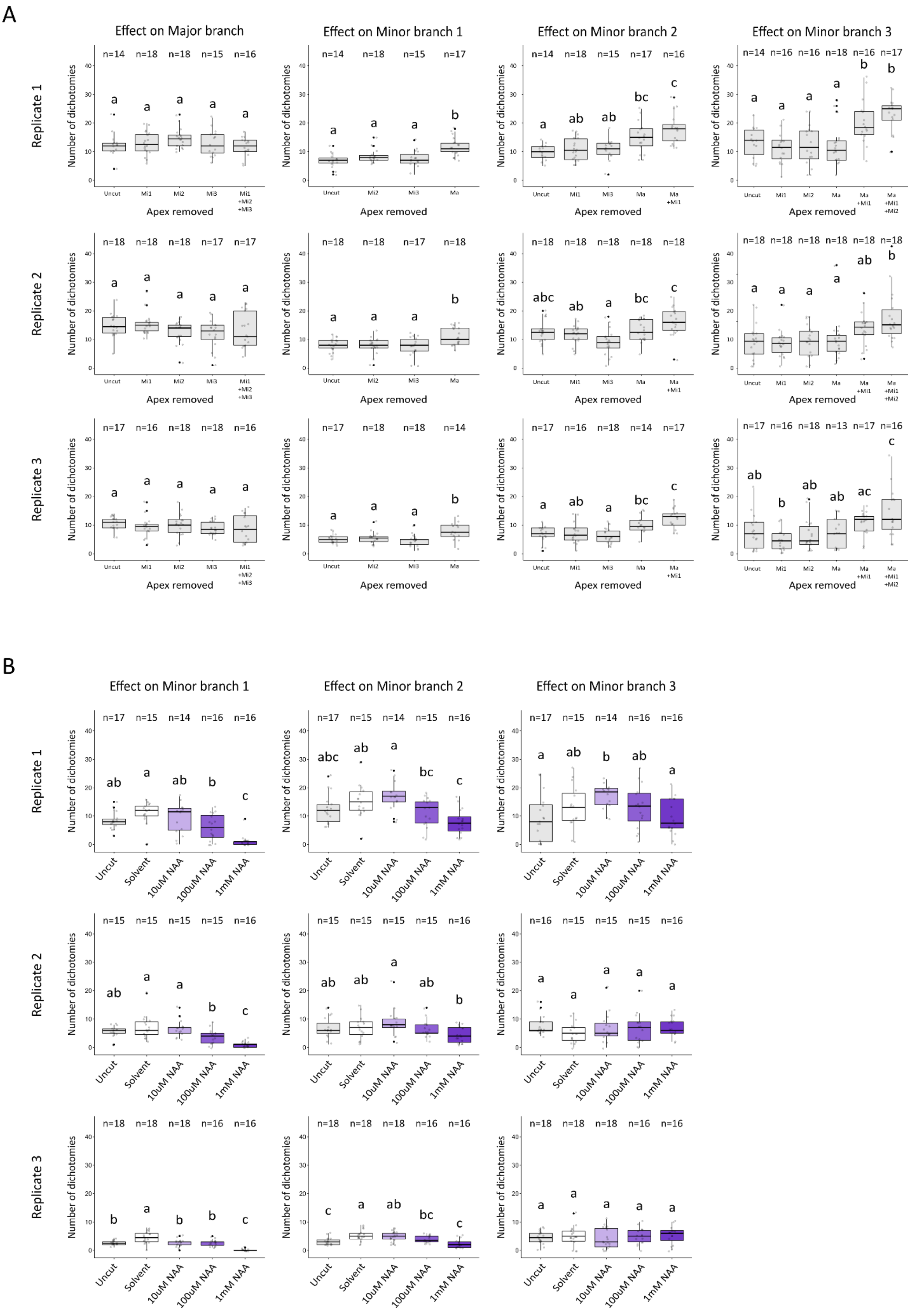
A
Effect on Major branch
Effect on Minor branch 1
Effect on Minor branch 2
Effect on Minor branch 3
Replicate 1
Replicate 2
Replicate 3
Number of dichotomies
Apex removed
Uncut
Mi1
Mi2
Mi3
Ma
Mi1 +Mi2 +Mi3
Ma +Mi1
Ma +Mi1 +Mi2
n=14 n=18 n=18 n=15 n=16
n=14 n=18 n=15 n=17
n=14 n=18 n=15 n=17 n=16
n=14 n=16 n=16 n=18 n=16 n=17
n=18 n=18 n=18 n=17 n=17
n=18 n=18 n=17 n=18
n=18 n=18 n=18 n=18 n=18
n=18 n=18 n=18 n=18 n=18 n=18
n=17 n=16 n=18 n=18 n=16
n=17 n=18 n=18 n=14
n=17 n=16 n=18 n=14 n=17
n=17 n=16 n=18 n=13 n=17 n=16
B
Effect on Minor branch 1
Effect on Minor branch 2
Effect on Minor branch 3
Replicate 1
Replicate 2
Replicate 3
Number of dichotomies
Uncut
Solvent
10uM NAA
100uM NAA
1mM NAA
n=17 n=15 n=14 n=16 n=16
n=15 n=15 n=15 n=15 n=16
n=16 n=15 n=15 n=15 n=16
n=18 n=18 n=18 n=16 n=16

Fig. S5. related to Figure 3: Data from apex decapitation and lanolin-auxin application replicates. (**A**) Graphs representing data from three experimental replicates of decapitation experiments using explants with three dichotomies and grown in soil for eight weeks. Either the major, minor 1, minor 2, minor 3 or a combination of these branches were removed, and the subsequent dichotomy rates recorded. In all graphs, boxes represent lower quartile, median and upper quartile. Lines represent spread of data and black points show outliers. One-way ANOVAs with Tukey tests for multiple comparisons were performed ($F(4,76) = 1.28$, $p = 0.28$; $F(3,60) = 9.83$, $p < 0.0001$; $F(4,75) = 9.94$, $p < 0.0001$; $F(5,91) = 8.95$, $p < 0.0001$; $F(4,83) = 1.73$, $p = 0.15$; $F(3,67) = 5.39$, $p = 0.002$; $F(4,85) = 6.7$, $p < 0.0001$; $F(5,102) = 5.19$, $p = 0.00028$; $F(4,80) = 0.99$, $p = 0.42$; $F(3,63) = 6.62$, $p = 0.00059$; $F(4,77) = 13.2$, $p < 0.0001$; $F(5,91) = 6.08$, $p < 0.0001$). (**B**) Graphs showing data from three experimental replicates for replacement of the major branch with lanolin impregnated with varying concentrations of auxin. Explants were grown for eight weeks on soil. In all graphs, boxes represent lower quartile, median and upper quartile. Lines represent spread of data and black points show outliers. One-way ANOVAs with Tukey tests for multiple comparisons were performed ($F(4,73) = 14.63$, $p < 0.0001$; $F(4,72) = 6.86$, $p < 0.0001$; $F(4,73) = 2.91$, $p = 0.027$; $F(4,71) = 12.95$, $p < 0.0001$; $F(4,71) = 2.76$, $p = 0.034$; $F(4,72) = 0.81$, $p = 0.52$; $F(4,81) = 24.77$, $p < 0.0001$; $F(4,81) = 10.54$, $p < 0.0001$; $F(4,81) = 0.35$, $p = 0.84$).

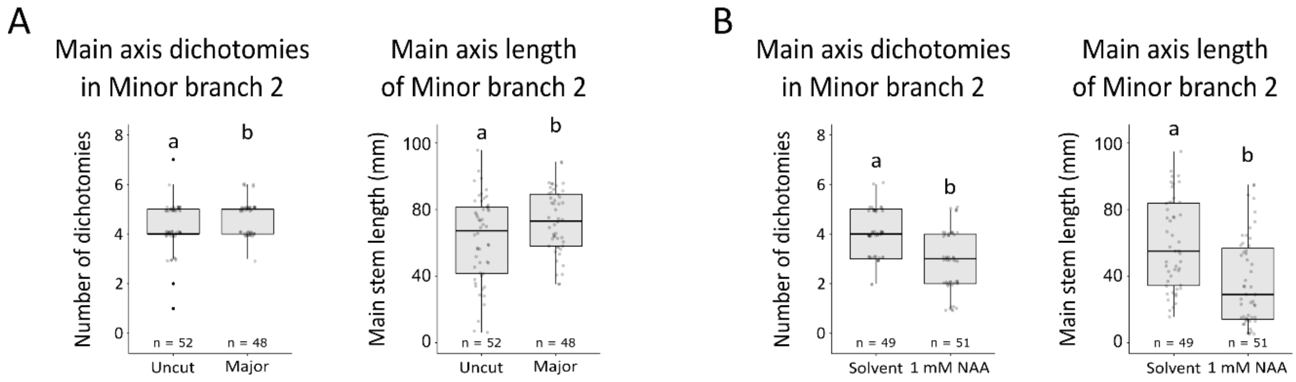

Fig. S6. related to Figure 3: Growth phenotypes of Minor branch 2 after Major branch decapitation and auxin application. (**A**) Both the number of dichotomies along the main axis of Minor branch 2 and the length of the main axis of Minor branch 2 increased after removal of Major branch tips. (**B**) Replacement of excised Major branch tips with 1 mM NAA reduced both the number of dichotomies and the length of the main axis of Minor branch 2. In all graphs, boxes represent lower quartile, median and upper quartile. Lines represent spread of data and black points show outliers. One-way ANOVAs with Tukey tests for multiple comparisons were performed ($F(1,98) = 5.15$, $p = 0.025$; $F(1,98) = 5.45$, $p = 0.022$; $F(1,98) = 28.53$, $p < 0.0001$; $F(1,98) = 17.64$, $p < 0.0001$). Data are from one experiment with n indicated on each graph.

Development • Supplementary information

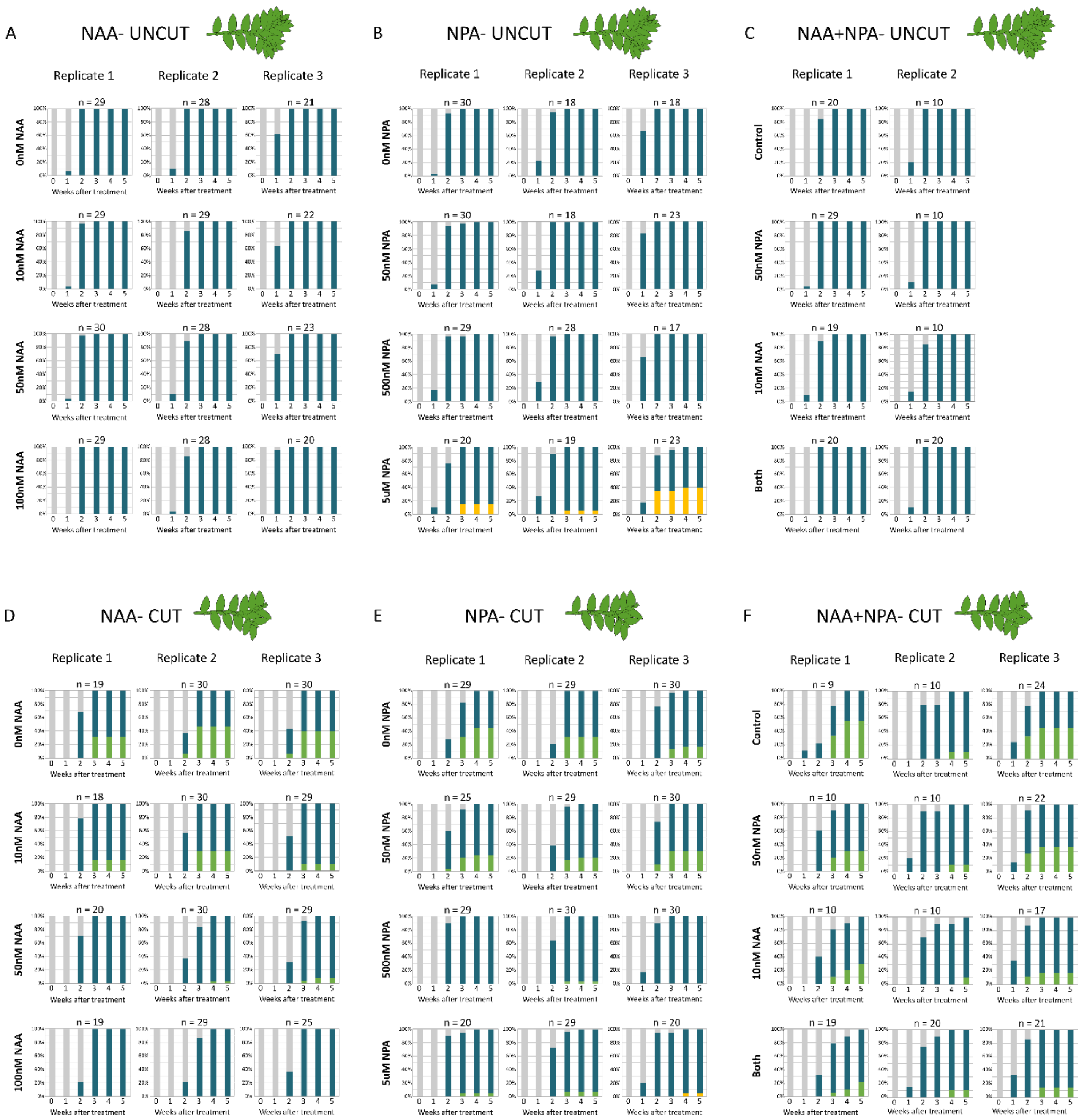

Fig. S7 related to Figure 4. Effect of surgical decapitations and pharmacological treatments on rhizophore vs angle shoot fate. (**A, D**) Graphs representing data from three experimental replicates grown in axenic culture with 0 nM, 10 nM, 50 nM or 100 nM NAA. Explants had 1 dichotomy and the apices were (**A**) left intact (Uncut) or (**D**) removed (Cut). (**B, E**) Graphs representing data from three experimental replicates grown in axenic culture with 0 nM, 50 nM, 500 nM or 5 μM NPA. Explants had 1 dichotomy and the apices were (**B**) left intact (Uncut) or (**E**) removed (Cut). (**C, F**) Graphs representing data from two/ three experimental replicates grown in axenic culture with 0 nM, 10 nM NAA, 50 nM NPA or 10 nM NAA + 50 nM NPA. Explants had 1 dichotomy and the apices were (**C**) left intact (Uncut) or (**F**) removed (Cut).

Development • Supplementary information

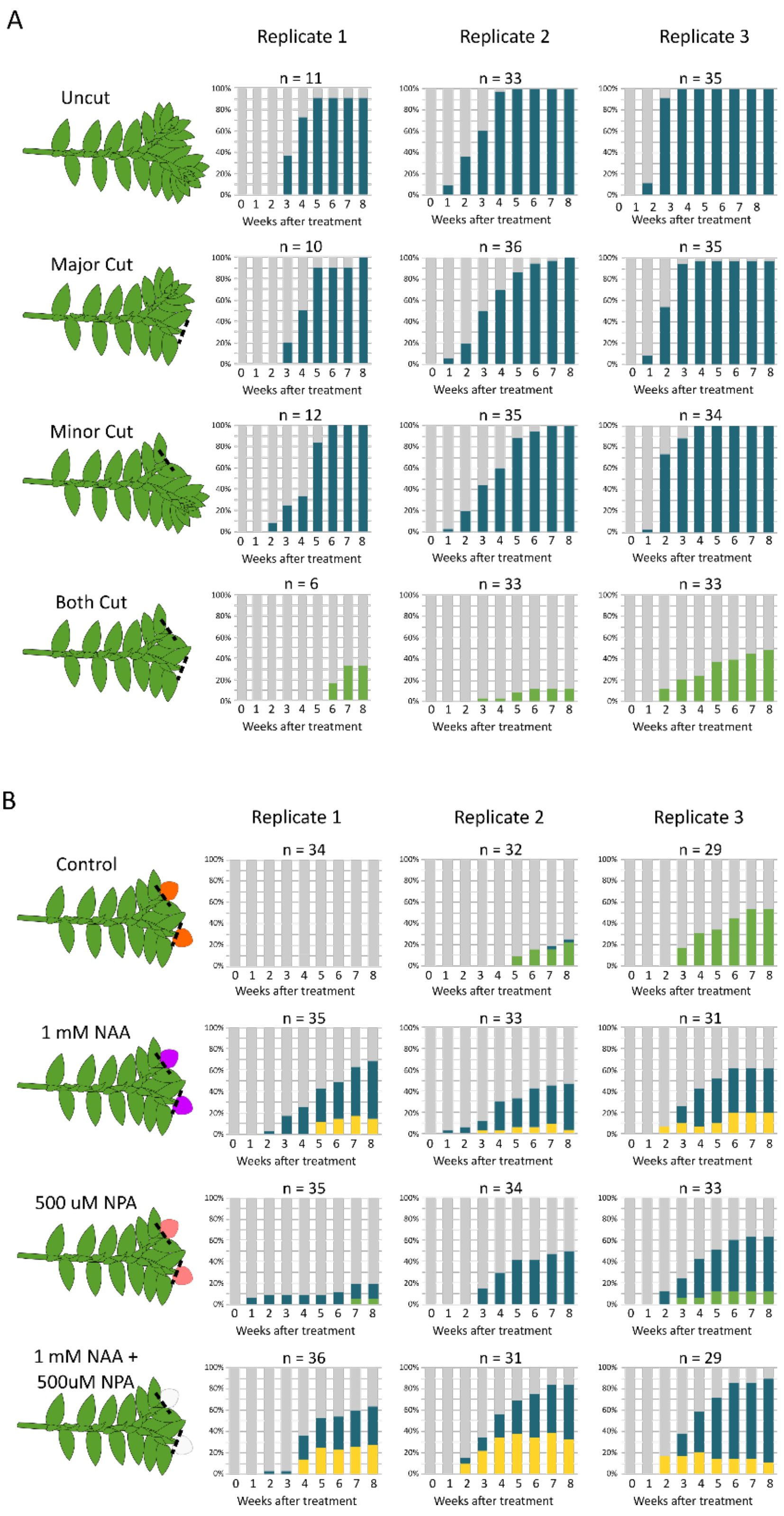

Fig. S8 related to Figure 4. Effect of surgical decapitations and pharmacological treatments on rhizophore vs angle shoot fate in plants grown on soil. (**A**) Graphs representing data from three replicates of decapitation experiments. (**B**) Graphs representing data from three replicates of experiments using decapitation and replacement of decapitated apices with lanolin paste containing 1 mM NAA, 500 μM NPA or a combination of 1 mM NAA and 500 μM NPA. In all graphs, grey shading indicates angle meristem identity, blue shading indicates rhizophore identity, green shading indicates angle shoot identity and yellow shading indicates callus.

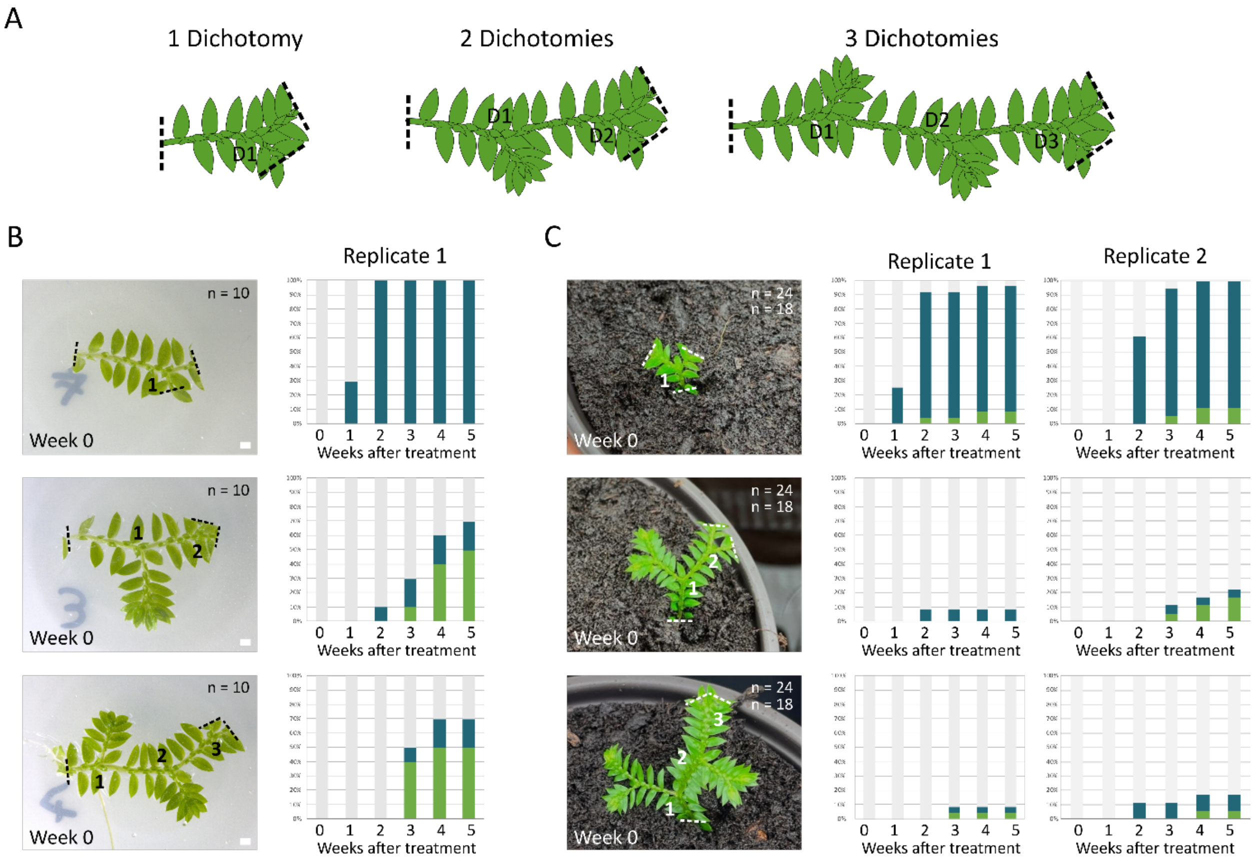

Fig. S9 related to Figure 4. Explant length influences angle meristem fate. (**A**) The apices of explants with either 1, 2 or 3 dichotomies (D) on the main stem were removed. (**B**) Different length explants were transferred to media and the identity of the organ produced from the angle meristem was recorded over a 5-week time course. Whilst explants with 1 dichotomy all produced rhizophores (blue), explants with 2 and 3 dichotomies produced fewer rhizophores and more angle shoots (green). N = 10. (**C**) The experiment performed in B was repeated twice on soil. Nearly all explants with 1 dichotomy produced rhizophores, whilst rhizophore identity was inhibited in explants with 2 and 3 dichotomies. N = 24 and n = 18 for the two replicates respectively.

Development • Supplementary information

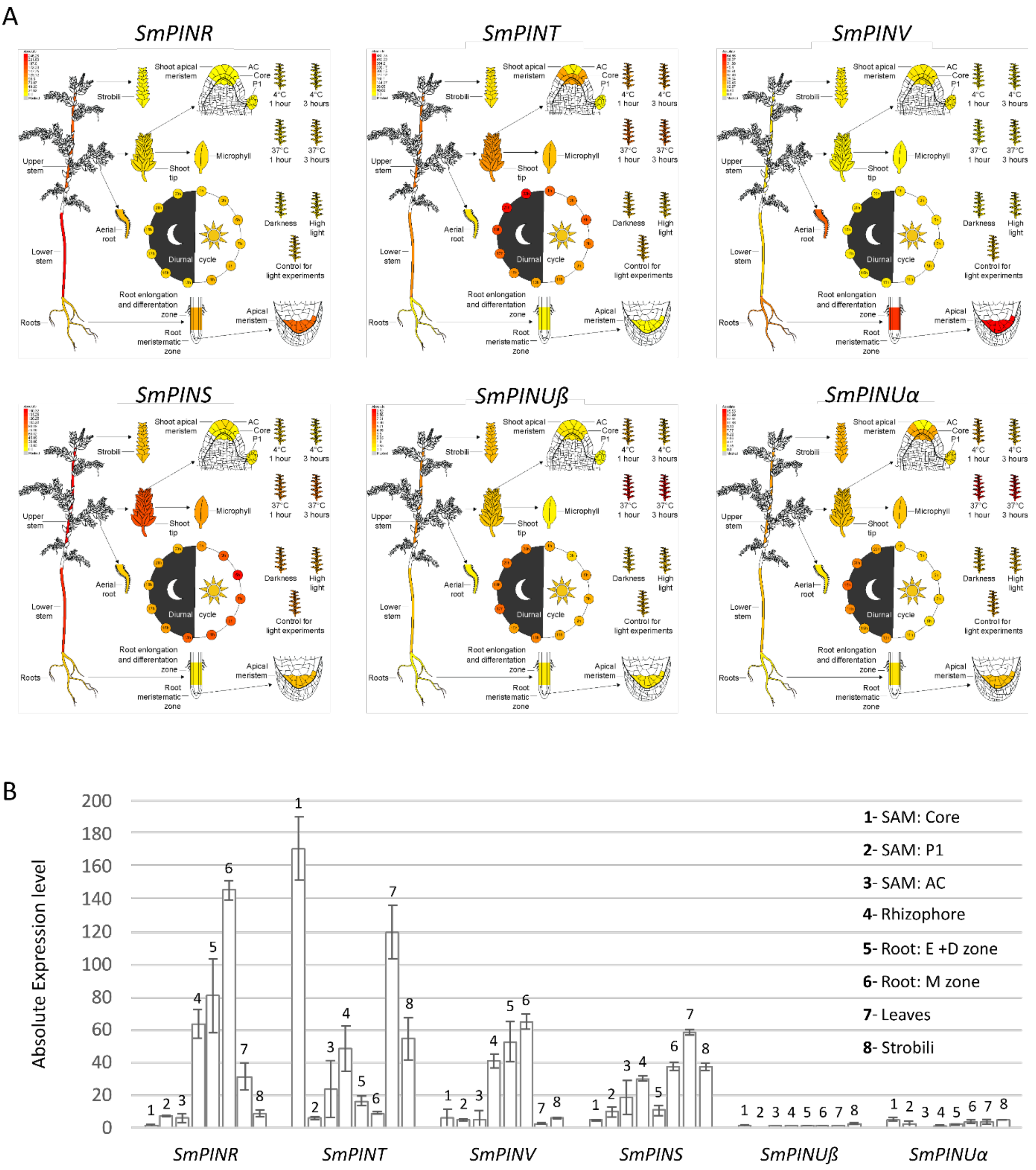

Fig. S10 related to Figure 5. *Selaginella moellendorffii PIN in silico* expression data. (**A-B**) *In silico* RNA-Seq expression analyses of *S. moellendorffii PIN* genes from the BAR *Selaginella* eFP browser (Ferrari et al., 2020). The same data are represented as heat maps (**A**) and bar graphs (**B**). *SmPINR, SmPINT, SmPINV* and *SmPINS* are expressed variably across different tissues. *SmPINUα* and *SmPINUß* are not expressed as highly in any tissue. *SmPINR* is upregulated in the maturation zone of the zoot; *SmPINT* is upregulated in the SAM core; *SmPINV* is upregulated in all regions of the root; and *SmPINS* is upregulated in the leaves.

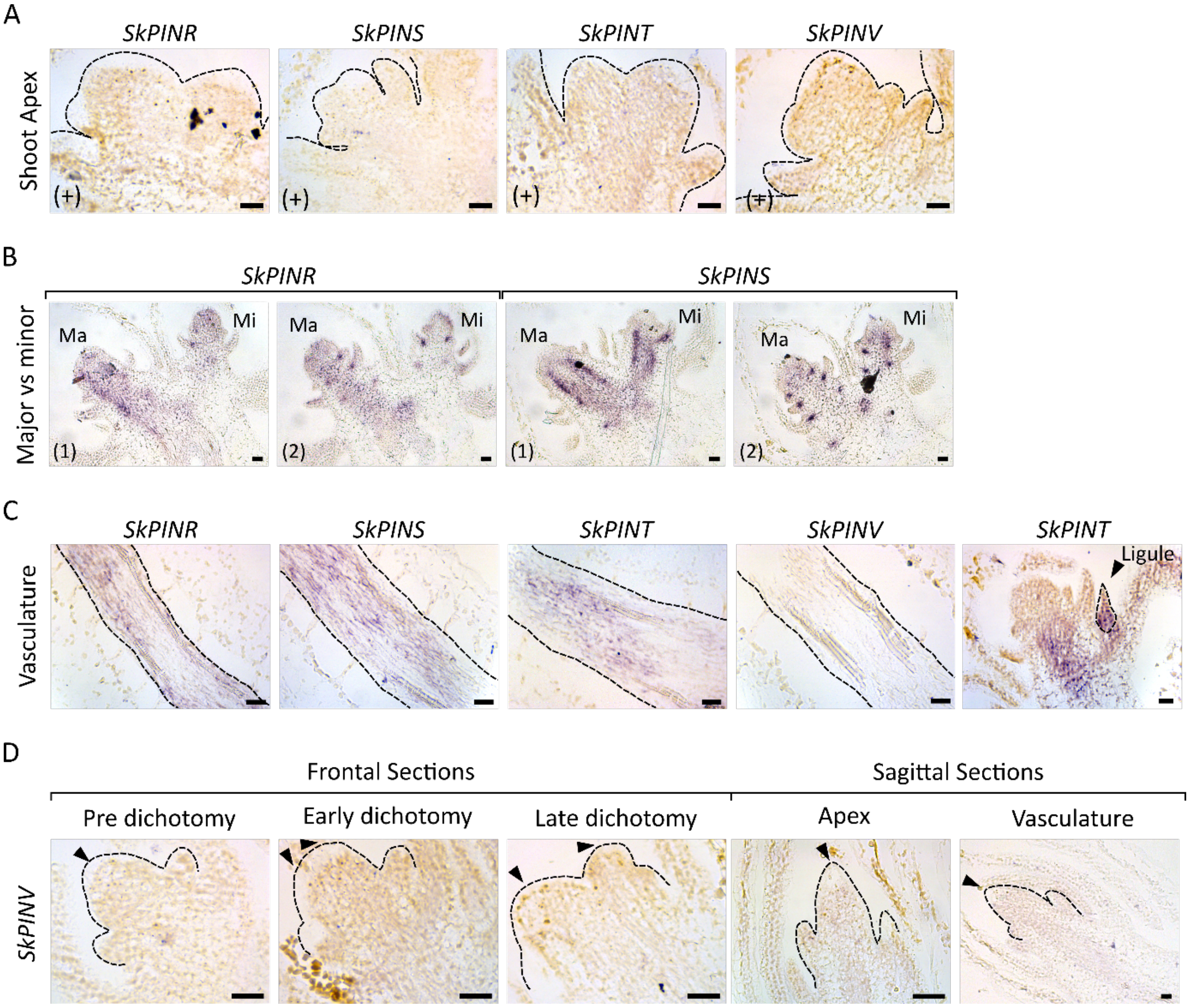

Fig. S11 related to Figure 6: RNA *in situ* hybridisation controls and further data. (**A**) Sense controls (+) for RNA *in situ* hybridisation of *S. kraussiana PIN* genes in the shoot apex during dichotomy. Scale bar = 0.02 mm. (**B**) There is no noticeable difference in *SkPINR* or *SkPINS* expression between recently separated Major (Ma) and Minor (Mi) branches. Two sequential sections are shown. Section (1) shows broad expression in the developing stem vasculature and section (2) shows discrete signal in developing vascular traces of leaves. Scale bar = 0.02 mm. (**C**) *SkPINR, SkPINS* and *SkPINT* were expressed in the vasculature. *SkPINT* was also expressed in ligules (arrowhead). Scale bar = 0.02 mm. (**D**) *SkPINV* expression was undetectable in frontal and sagittal sections of the shoot apex. Arrowheads = shoot apex. Scale bar = 0.02 mm

Development • Supplementary information

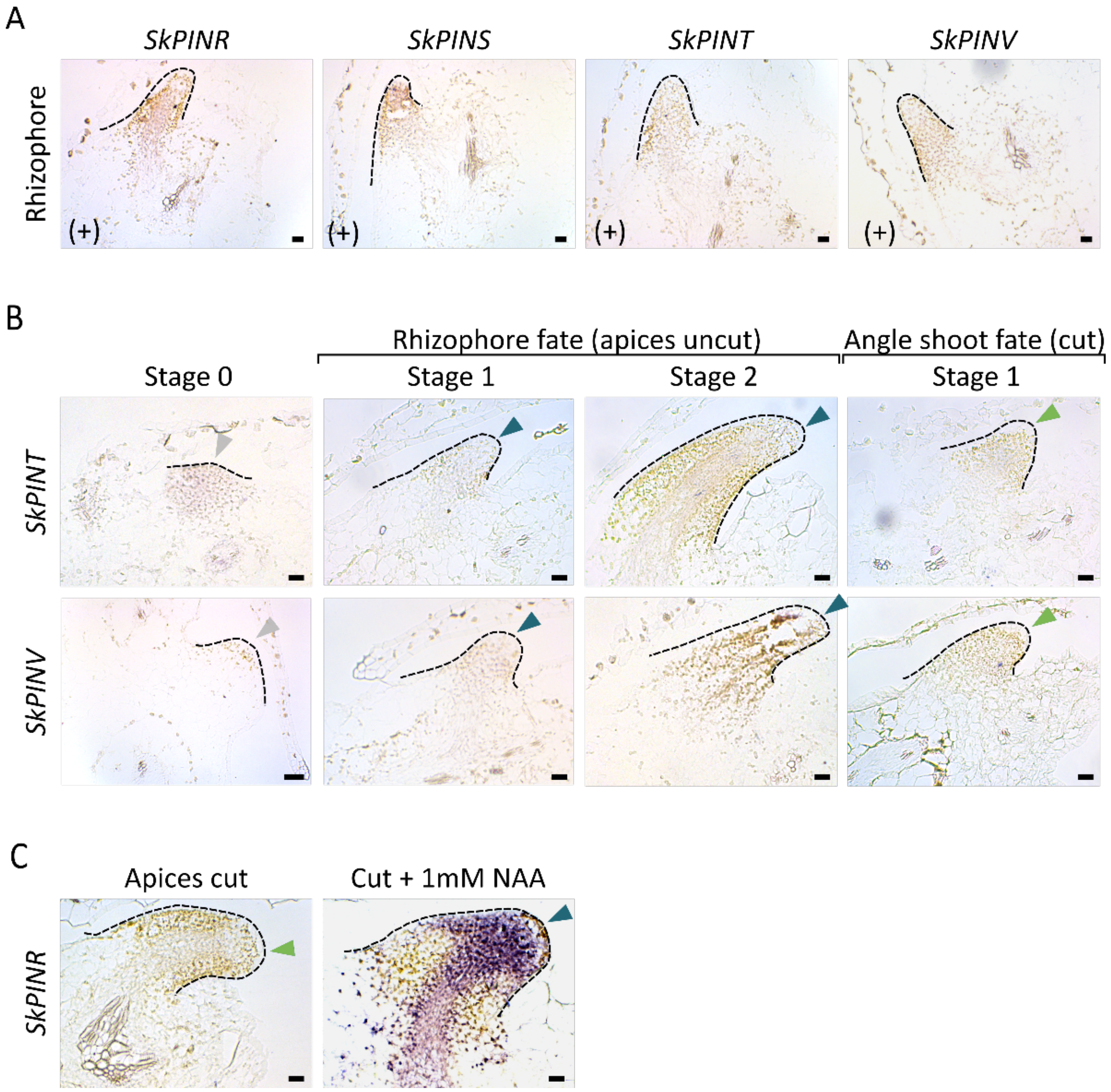

Fig. S12 related to Figure 7. RNA *in situ* hybridisation controls and further data. (**A**) Sense controls (+) for RNA *in situ* hybridisation of *S. kraussiana PIN* genes in the rhizophore. Scale bar = 0.02 mm. (**B**) *SkPINT* and *SkPINV* expression in the angle meristem and rhizophore was undetectable prior to and 1 week after apex removal. Grey arrowhead = angle meristem, blue arrowhead = rhizophore apex, green arrowhead = angle shoot apex. (**C**) *SkPINR* expression was low 1 week after apex removal, but dramatically increased if the apices were replaced with lanolin paste + 1 mM NAA. Blue arrowhead = rhizophore apex, green arrowhead = angle shoot apex. Scale bar = 0.02 mm.

Development • Supplementary information

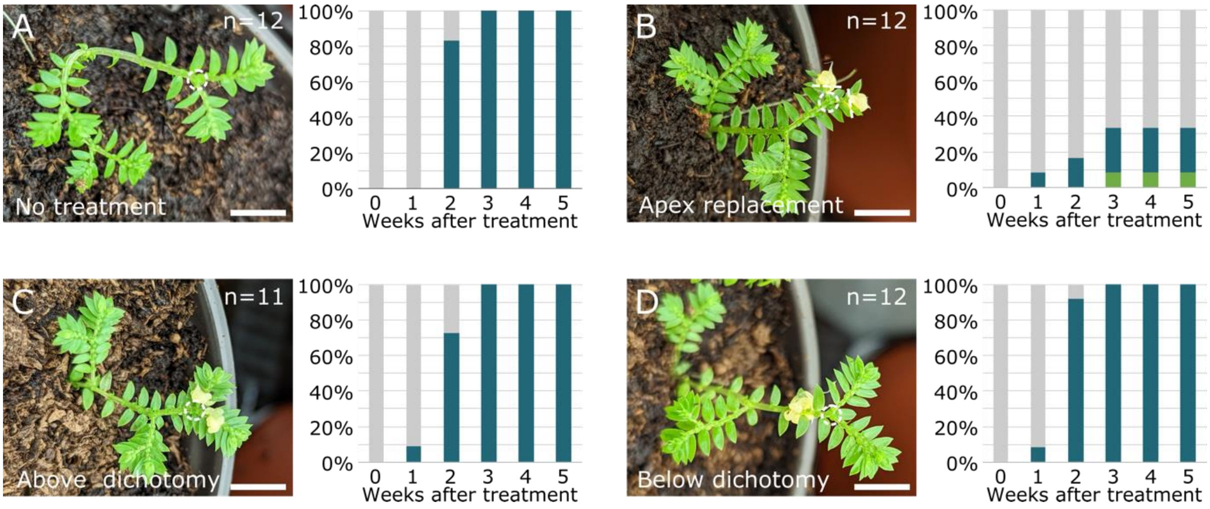

Fig. S13. A lanolin paste containing auxin transport inhibitors and applied around the stem had no effect on angle meristem identity. *S. kraussiana* explants with 3 dichotomies were either (A) untreated or (B) both apices were removed and replaced with 500 µM NPA + lanolin. As in Fig. 4, the angle meristem (white dashed circle) produced rhizophores in untreated explants, or rhizophores or angle shoots in treated explants. To attempt to block auxin transport in stems, 500 µM NPA + lanolin was applied around the stems (C) above or (D) below angle meristems. These treatments showed no effect, likely due to poor tissue penetration. Blue = rhizophores. Green = angle shoots. Scale bar: 5mm.

Development • Supplementary information

Table S1. Primers used in this study.

**Name of primer:**	**Sequence 5’-> 3’:**
**CDS Cloning primers:**	
SkPINR Forw	ATGATCAAGCTCGGGGAC
SkPINR Rev	CAAGTAAGAACACCCGAATGAAG
SkPINS Forw	ATGATCCTCGCATACGGCTC
SkPINS Rev	CCTGAACTATAGAAACGTGAAGGC
SkPINT Forw	ATGATCAGCGGGGAGGAT
SkPINT Rev	GCCGAGGAGCACGTAATATATC
SkPINV Forw	ATGATTACGCCCAGCGAG
SkPINV Rev	GGCCCAACAGTACGTAGTAG
**3’ UTR Cloning primers:**	
Q_T (Frohman, 1990)	CCAGTGAGCAGAGTGACGAGGACTCGAGCTCAAGCTTTTTTTTTTTTTTTTT
Q_O(Frohman, 1990)	CCAGTGAGCAGAGTGACG
Q_I (Frohman, 1990)	GAGGACTCGAGCTCAAGC
SkPINR 3’RACE GSP1	CAACTTCACCAATGCCGACG
SkPINR 3’RACE GSP2	GTCCAGTGCTGGTTTAGGG
SkPINS 3’RACE GSP1	GATGATGATGATCCCCAACCATCC
SkPINS 3’RACE GSP2	CAGAGCAAGGTCGGCTCAAC
SkPINT 3’RACE GSP1	ATGATCAGCGGGGAGGAT
SkPINT 3’RACE GSP2	CATCCTGAGATCGCCCATG
SkPINV 3’RACE GSP1	GGGCTCTTCGTACAATCTTCC
SkPINV 3’RACE GSP2	CAATGCCATCCACAGCAGTCTTG
**Probe primers:**	
SkPINR TM Probe 5'	ATGATACTCGCATACGGCTCAG
SkPINR TM Probe 3'	GACGTGGATCTTACCATCGTC
SkPINR Loop Probe 5’	GAGCGGAGATCTACTCGATGC
SkPINR Loop Probe 3’	GCTCCGTAGTGGAACTGGTG

Development • Supplementary information

SkPINS TM Probe 5’	ACGTCAAGATGCCGGACATAG
SkPINS TM Probe 3’	TCTCAACTTGGGGGTGCTGT
SkPINS Loop Probe 5’	GCGCCGACATCTACTCGATC
SkPINS Loop Probe 3’	GTGGTGCTGCTGCTGTG
SkPINT TM Probe 5’	ATGATCCTTGCATATGGCAGCG
SkPINT TM Probe 3’	GACATGGATGCGACCATCAC
SkPINT Loop Probe 5’	GCGCTGAGATCTACTCGCTTC
SkPINT Loop Probe 3’	CTTGATGAATCTTTGGCGTGG
SkPINV TM Probe 5’	ATGATCCTCGCCTACGGCTC
SkPINV TM Probe 3’	GACGTGGATCTTACCGTCG
SkPINV Loop Probe 5’	GCAGCAACGACAACGCTTC
SkPINV Loop -+Probe 3’	CGTTGTTGCAGGTGATTCG

Development • Supplementary information

Table S2. ***Selaginella kraussiana*** **PIN gene sequences**

… = Unsequenced Grey = Exons White = Introns Underlined = 3' UTR

PINR

…ATGATCAAGCTCGGGGACTTGTACAATGTGCTCAGCGCGGTGGTACCACTCTACGTCGCGATGATACTCGCATACGGCTCAGTACGCTGGTGGCGTATC
CTCACGCCGGAGCAATGCGGGGGCATCAATCGCTTCGTCGCCATCTTCGCAGTCCCTCTCCTCTCCTTCGAGATCGTCTCGGGCAACAATCCCTACAAGAT
GAACCTCAAGTTCGTGCTCGCAGACGGGCTCCAGAAGGTGATCATCCTCGCAGCGCTCGCAATCTGGGCTCGCATCAGCAGCGCCCGGCAACGGGCAGC
TCCGATGGATTCCTCGAGCAGCGGGAGCGCCACGAGCAGCAGGCTCGACTGGGTGATCACGCTCTTCATGCTCTCGACGCTCCCGAACACGCTGGTCATG
GGCGTCCCGCTCCTCGCAGCGATGTATGGCCGCTACCCCGGCTCGCTCGTCGTCCAGGCCGTCGTTCTACAGTGCGTCATCTGGTATACGCTCCTCCTCTTC
CTCTACGAGTACCGCGCAGCGCGGATCCTCATCCGCGAGCAGTTCCCGGACACCGCCGCCTCGATCGTCTCCTTCAAGGTCGACTCCGACGTGATCTCCCT
TGACGGACGCGACCCCGTTCAGGCGGACGCCGAAGTCGGCGACGATGGTAAGATCCACGTCGTCGTGAGACGCTCGGTATCGCTCTCCTCGCCGCCGCA
CCGCCGGTCGTCGAGAGCGGCTGCTGGGATCACACCGCGGGCGTCCAACCTCACGGGAGCGGAGATCTACTCGATGCATTCGTCTCGCAACTTGAGCCC
GCGGCACTCCAACTACGGCGGCGGCGCCGCCAACACCCGCGACGACCTTCTCTTGCTCCAGCGTCCCTCCAACTTCACCAATGCCGACGTCTACTCGTTGC
ATTCCTCGCGGGCGCCCACGCCGAGGACGTCCAACTTCAATGACGCCGCTGGTGCGCCGCTACCGCCGTGGCTCAACAACAGTCCTCATCACCATGGTGCT
GCTGCCAATGGTAACGGCAGTGGTAACCAGCAGGATCATATGTTCGTTTGGAGCTCCAGCAATTCGCCGGTCTCGCATCCTCTTCATCAGGCGGCTGCCGC
CGCTGCTGCTGCTGCAAGGCATCACCAACAATCGGGTATGTATATCTTCTTTCTTCGTATTGGGAACGTATGTTCTTATGTTGAGTTCATATACATACGGTCT
ACAATGTGTTCATTAGTGAGAACGTATATTCTTATATTAAGGTCATTGTTTTTTAATTATTTTTGGGTCTTGTTGTATTGATTTTCATATTATTTGGTCATTTAA
GACCTGAAATTGAGAGTATGTGTATACTTCCATACGTAGAATGTACATGTACAACGTTAAGGTCCCTTTCAACCAAAGACTACATTCTTGAGCTTTTCATTC
ATTCAACATATTGTGTGTGTGTGTGTATGTTCCTTTCGTGGCATTCATTGAATTCCTCCTCTGCCATAGTTGGCTATACACCAAAAATCTGTTGTTTCTGATAT
GAGCAGAAATCATTTGAGCAAGTGTGGCTCCAAATCTGGGGCACATTTTTCCTGCAAGATGTGTCCGTCCAGCAACTTGCATGCTACATAACTGTGCCTTC
CATTCTTCAGACTTCTTGTTCCTCAAGAACGTTACGTCTGACCTTGTCTATCAAATGGATTTTGCTTCTTTGCAGCTCCTGCTCGGTTTGACGACAACAGGAA
CGAGTTCAGCTTCGGGAATCCCGTGATCAGCTTTCGTGACGACTCGATCCTTCCTCCAGCAGACAAGGAGGCATGTCCAAGCCTCACAAAGATGGGTTCCA
CCAGTTCCACTACGGAGCTCGCACCCAAAGCCGGGCAACACATCGATATGCCACCAGCCCGGGTCATGACCAAGCTCATTCTCAACATGGTCTGGAGGAA
GTTAATCCGGAACCCTAACACGTACTCCAGTTTGATCGCTGTTGTCTGGGCTCTCATCTCATATCGGTATGTTTATATGTTAAACCCTAAACCGTGTATTAAA
TATTTCGGGGTTTTTGTATCAGGTGGAACCTCAAAATGCCTGCTATTGTGGACAAGTCCATCACCATCTTGTCCAGTGCTGGTTTAGGGATGGCAATGTTCA
GTTTAGGTATGTACCATATACTTTTGAGGTGTCATTTTCTGGTATGATTTCAGGTCTTTTCATGGCCTTACAAAAGAGGCTTCTGGCTTGTGGAACACCTCTT
ACATGCTATGGTATGCTTGTACGATTTGTCACAGGTCCTGCAGTGATGGCTGCTGCTTCCATTGCTGTTGGGCTTCGTGGTGTGGACCTCAAGGTCTCAAT
AGTTCAGGTACGTACGTTACTGTGAATATCGGTCGAGTTTGGAAATGTTGATATGTTGATTTTCTTACATCAGGCGGCGCTTCCACAAGGAATTGTGCCTTT
CGTTTTTGCTAAAGAGTACAACGTTCATCCTGATATCTTGAGCACAGCGTAAGCATGCATTGTGGTGACCACGGAAATTGAGGTGTTTTTCTTGCTTTTCAG
GGTCATCTTTGGCATGCTGGTGGCGTTCCCGATCACGCTCTTGTATTACGTGTTACTCGGTTTATGATCGCAATTGAACAAACAAGCACCTCAGTTTTTGCC
CCCCCTTTTGATGCCAGCCAGACTCCGAACCTTCATTCGGGTGTTCTTACTTGTTTTGCAAACACCACAAGCTTGCCATAAGTTCGTTGTGATTTAGTTTTAA
TATATACTAAATTATT

PINS

…ATGATCCTCGCATACGGCTCAGTCCGCTGGTGGCGGATCCTCACGCCGGAGCAGTGCGGGGGCATCAACCGCTTCGTCGCCATCTTCGCAGTTCCACTT
CTCAGCTTCCAGATCCTCGCCGCGAACGACCCCTACAAGATGAACGCGCAGTTCGTCGCAGCGGACGCACTCCAGAAGATCGCCATCCTCGCCGTGCTCG
GCCTCTGGGCGCGCTACTCCTCCAACGGGAGCATGGAGTGGATGATCACACTCTTCATGCTCGCCACCCTCCCCAACACGCTCGTCATGGGAATCCCACTC
CTCGAAGCCATGTATGGCATCCAATCGGGCGACCTCGTCGTGCAGGCGGTCGTCCTCCAGTGTATCATCTGGTACACGCTCCTCCTCGTCTTCTACGAGTAC
CGCTCCGCGCGCAATCTCATCCGAGAGCAGTTCCCCAAGACCGCCGCTTCCATCGTCTCCTTCCGTGTCGATTCCGACGTCGTCTCGCTCGACGGAGCCGTC
GACCCCATCCAGGCCGACGCCGAGGTCGGCGACGACGGTAAGATCCATGTCACCGTCCGCCGCTCGACGTCGTCCAGGCACTCGGCGATGTTCTCCCCAT
GTCGGACGTCCAAGGTCGGAGCCGGCGGCGGTGCCGGGCTCACACCTCGGCCCTCCAACCTGACCGGCGCCGACATCTACTCGATCCATTCGTCTCGGAG
TCTGACGCCGCGAGAATCGAGCTTCAACCACTCGGACTACTACTCGATGATGATGATCCCCAACCATCCGGCGCCGCCATCGCACTCGCACTCTCACATGT
TCGGGCGGAGTCCCAGACAGTCCAACTTCGGGGCGTCCGATGTCTACTCGCTGCACTCGTCTCGTGGTCCGACTCCCCGAACTTCCAACTTCAACCTCGAC
GATGCCATCCAGCCGAGGCTCATGAGCTTCGTTCCCGCCACTACCAACGTCGCTCAGCCTCCGCCGCCACCGCCGCCGCCGGTCCCGCCTCGGAACAGCAA
TGCAGCCGCGAATGCAGCGGCGGCGGCGGCGGCTGCTTCCGCCGCCGCTGGTGGAAGCGCCGCCAACAACAAGGACCTGGTGCATATGTTCGTGTGGA
GCTCCACGGGATCGCCCATCTCCGAAGGCAACGTGCAGATGCTCAACGACGACTTCCGCAACAAGGACGTGCGTCTCATGGTCGCCAACGACGATAACCC
GGCGTCTCATGGTGCTACCACTACCACCGGTACGTCCCAACTTCTCTCGTATAATCATCTCACGGAGGTTCCCCCACAGATGGAGTCTACGAGCGGGACCG
TCGGGACGAGTTCAGCTTCGGACACGCCGGCGTCGCCGACGAGCAGAGCAAGGTCGGCTCAACCTCAACCACGGATCCACAGCAGCAGCACCACGACGT
CGAGGGTGGCGGCGGTGCTCCAGCGGAGATGCCGGCGGTGAGCGTCATGACGAGGCTCATCTTGGACATGGTCTGGAGGAAGCTAGTCCGCAACCCGA
ACACGTACTCGAGCTTACTCGGCCTCGGCTGGGCCCTCATATCTTTCAAGTCAGTATCTTGTTCGAGTCCGAGTCCGAGTCCGAGTCCGAGACTAACCTCGT
GTATGTTTGTTTACTTAGATGGCACGTCAAGATGCCGGACATAGTCCACAACTCGGTGCTCATCTTGTCCAAGGCCGGGCTCGGAATGGCCATGTTCAGTC
TCGGTACGTCGTAGTCGTGTACTCGTTCTCGGACAGGTCTTTGATGGATCGTACTCGCATTCCTTGTGTGTGTTGCTCCAGGCCTGTTCATGGCGCTGCAGA
AGCGGATCCTCGTGTGCGGTACGTCCCTGGCCGTCTTCGGCATGGTGGTCCGCTTCATCACGGGCCCCGCCGTCATGGCCGCAACCTCCATCGCAGTCGG
CCTCCGGGGTGTTAGCCTTCACGTTTCTATAGTTCAGGTATAGACTCGCATTAAATGCCCGATTTTTCATTGTTTCCTACAGGCAGCTCTGCCTCAAGGCATT
GTCCCCTTCGTGTTCGCAAAGGAGTACAACGTACACCCCGACGTCCTCAGCACTGCGTATGTCTATACTGAAACCGTAGAGTAGGCTCTTAACGGACATAG
TATAGGCTTTAAACAACCCCGGTTTAGGGTTTAGGGTTAGGGTTTAGGTCTAAACTAATGTTGATGTTTTTTCAGGGTCATTTTCGGCATGCTGGTGGCCTT
CCCCATCACGATGCTCTACTATGTGCTGCTCGGCTTGTAAAAAAATGACTGCCTGCCTGCCTATATATCCTGCCTTGCTGGTACTCAATATGGAGAGGACGA
TGATGGTACCATCCTTCCACCACCACGGAAGACACAAATTAGTTTTTGCATATGCTGCTGAACTTGCAGTGCAGCAAAGCAGCAGCAGCCAGACAGCACCC
CCAAGTTGAGAGAAGACGACTGTTTCTGCTGCTCTGTTAGGCCCCCACTCAGTTTTTTTTTAATCCCAAGTGCAGTATTCCTCTGT

Development • Supplementary information

PINT
…ATGATCAGCGGGGAGGATTTCTACAACGTTATGGCCGCCGTGGTGCCCCTCTACGTTGCCATGATCCTTGCATATGGCAGCGTTCGATGGTGGGGGCTC<br>CTCTCACCCGATCAATGCTCGGGGATCAACCGTTTCGTGGCACTCTTCGCAGTCCCGCTACTTTCATTCCAGATCATCTCCAAGAACAACCCATACGAGATG<br>GATCCTCAGTTCATAGCTGCGGACGCAATGCAGAAAGGCCTCGTCCTGGCAGTGCTAACCGCATGGTGCCGCTGGGACACGCGACGAGCGACCTTTGAAT<br>GGGTAATCACGCACTTCATGGTAGCAACGCTCCCGAACACGCTGGTCATGGGAATTCCCCTCCTGGGAGCAATGTATGGCAAGCACGGGGCTCTCGTAGT<br>CCAGGCCGTAGTCCTCCAGTGCATCGTATGGTATACGCTCCTCCTCATACTCTACGAATACCGTGCAGCCAAGCTGCTCATCCTCGAGCAATTCCCGGCCGG<br>AACCGCCGCTTCGATCGTGTCTTTCAAGGTGGACTCGGACGTTATATCCCTCGATGGACGTGAAGGAGCAGTCATGACGGAAGCCGAAGTCGGAGGTGA<br>TGGTCGCATCCATGTCAAGGTCCGGAGATCCACTTCCTCGGCCCCGAGGTTCTCCTCAGCATCCTCCGTGCAAATGCAACTCATGAACTCGTCCCAGAATCC<br>TCATCTCTTCCACCAGAACCATCTCCAAATGCCGCAACAATACGGTGGTGCCATCCTGAGATCGCCCATGGCTTCCATGTTTTCGTCCAAAGCTGGAACCCC<br>GCGCCCCTCCAATCTCACGGGCGCTGAGATCTACTCGCTTCAGTCGTCTCGCAATATGACACCCCGTGAGAGTAACTTTGGCCGTGGTGATCTTTATCTCAT<br>GGGTGGTGGTGGTGGTAGCGGCGCCGCTACCATGTTCGCGCCACGTCATTCAAACGTCACCACGGGCCTCGAGAATGGTGCCATGGCGAGTGGAACAGG<br>GGACCACGCTCTGGGAGCGTATTCCCCGTCTCCCGCCATGCAACTTCCGAAGAAAGTGAGTACTACCACCGGAAACGCTGGTGCTACCGCCGCCGGCGGT<br>AGAGCTTCCGACTACGACGCACGTGAGCTACATATGTTTGTCTGGAGCTCCAGTGCGTCACCGACGTCAGAACGGGGTGGCGTCCACGTCTTTGGAGGCA<br>CTGACGACGGTGTTGGCAAAGCTGACGGCACCAAGGAGTCCACAAAGCTTGTGGTTGTCGCAGACGATGCTCATGGCGATGGTATGTGGTTTCATCTTGG<br>ATTTCATGCATGGAAGTTCATCTCGCGTGTGCTGCTTTCAGATTCTTCACGAGATCATCGCGTAGAGTTCATGCAACCAGAGCAGCAGCCGCAGCAAGATT<br>TCAGCTTTCCTGGGAGCGAAAGAGCAGACGACAAACTGGTGGAACCGGGTTCTAACAAGCATGACGCAAGCTCTACAGAGATAACCACGCCAAAGATTC<br>ATCAAGTTAATGGAGGTTTAGAAGGGCTTAACCTCAACAATGGCAGCGATAATGGAGCACTGCCTCCAGCTTCAGTCATGACCAGGCTCATTCTCAGCAT<br>GGTTTGGAGGAAGCTCATCAGAAATCCAAACACTTACTCCAGTGTCATTGGTCTCGTCTGGGCACTCATCTCGTTCAGGTGTGTATTAGTTCATGATTATAG<br>TTCGTGTTTAGCCTCTGGAACGTTAATACGATCCGTGTTAGATGCTTCTTTGCAGATGGAACATCAAAGCTCCCAAGATCATCGAGAAGTCCATCACCATTC<br>TATCTGATGCTGGTCTTGGCATGGCTATGTTCAGCTTAGGTACTTGAGCATCAAAAAGCTCGTTTTTTTCTGAACTATTTTTTCACAGGCTTGTTCATGGCAC<br>TACAATCAAGACTATTGGCTTGTGGAACTTCCATGGCCTTGTTTGGGATGCTACTTAGGTTCTTTTGCGGGCCTGCTATCATGTCGGTAACCTCCATAGCCA<br>CTGGTCTGAGGAGTTTGGATCTTCGAGCTTCCATTGTGCAGGTAACGGTGCTACTATGGTTTCTAGCGTTAACGTGGTACGATGTTTACGCAGGCAGCACT<br>TCCCTCTGGAATTGTTCCCTTCGTCTTTGCGAAGGAATACAATGTTCACCCGGATGTTCTTAGCACAGCGTACGTTCTATATAGAAATTTTACTCGTTACTGA<br>TTTACTATCACTTTTCAGTGTCATTTTTGGCATGCTGGTGGCTTTGCCTATTACGCTGATATATTACGTGCTCCTCGGCATATAAAGATTTTGATTCATT
PINR
…ATGATTACGCCCAGCGAGTTCTACACCGTCATGACGGCGGTGGTACCACTCTACGTTGCAATGATCCTCGCCTACGGCTCAGTCCGATGGTGGAAGCTCT<br>TGACGCCATCCCAATGCTCTGGTATCAACCGCTTTGTTGCAATCTTCGCGGTACCCCTCCTCTCATTCCAAATCATCTCCCAAAATGATCCTTACCAAATGAA<br>CCCCAGGTTTGTAGGAGCAGACATACTCCAGAAGTTCATAGTTCTAGCTGCTCTTGGAGTCTGGAACGCCTTTAGAAACCACCGGCAAGAGAATCCAAACT<br>CGAGTCTGGATTGGACCATCACCCTCTTCATGGTGTCAACGTTGCCGAATACACTTGTTCTCGGAATACCTCTAGAGGTTGCAATGTACGGTAAGAAGCCA<br>GCAGCTCTTGTGGTCCAAGCAGTGGTACTCCAATGCATCGTATGGTATACCCTCCTCCTCTTCCTCTTCGAGTACAGAAGTGCCAGAAAGTTGGTACTGGA<br>GCAGTTCCCAGGACCTTCAGCCGCCAACATTGTCTCATTCCGCATCGATCCCGACGTTGTCTCCTTGGAAGGAGAACAAATCACGAGTGAGGCTGAAGTTG<br>GAAACGACGGTAAGATCCACGTCCAGTTGAAAAGGTCGCCCGTACCATCTTCTACTTGTTGCAAGTACCTTCCGAGATCTCCGACGTCTCAGTCCTCGAAA<br>GCTGCTACTCCACGGGCCTCTGACCTCACTGGTGTTGAGATCTACTCGCTACAGTCGTCGAGGAACATGACTCCCACGGGCTCTTCGTACAATCTTCCGGC<br>AGCAACGACAACGCTTCCTCGTCCTCGGAGCGCCAGTAACTGTGTCATTCCCGACACCAGCACCGCCTCGAACCATTCTTCGAATGTCCACACCCCTCGAG<br>AGTTGATCATTCCCGACTCTCTGGTCCAAGATTTACCCGCTCGTAACTATCGTTCGCCTCCCCCACGAGGAGCAAGGCTCGATCAACTGGATTTCCAAAAGG<br>CCGCAAGGAGTAAACTCGAGAAGCTGTTCATTCACAATGAGGCATTCTCTCCGATGCGGCTGGCCGAAGCCACCAAGAAAATGGACAGCTACCAATACGC<br>GGATGCTCAAGCTCGAGACCTTCAGATGCTTGTGTGGAGTTCTAGCACATCCCCTGCATCCGAGAGAGGGTCTATAAGTGTAGAGACCAAGAGGTTGACA<br>CCTTCCAAACCCCGGAGGCACTTGTCTATTGAAGGTACGATTTTTCTCGTTGCATGTTGCTTAATTCCATCTCATCCAATTTTTGCAGTTCCGATTACCTCAG<br>ATTCTACACCACTGCATCCCTCCTTGCAACACGACTTCAGCTTCGTAGACAACGTCCTGTTCCAGCGGGGTGGAGAAACCCCGGTGGATACCAAGATTAAA<br>ATCGAATCACCTGCAACAACGAAATTCCCCTTCACTCCACCGAAGGTTATCGACAATGTACCGACTACAATGCCATCCACAGCAGTCTTGACAAGCCTGATC<br>TTGAGAATGATGTGGAGGAAGCTTGTTCGGAATCCCAACACCTATGCAAGTTTGCTTGGACTCGCCTGGGCTCTAATCTCCTACCGGTACCATCTAAGATG<br>CGTTTTTTTTCTACTAACTCATGGATTTTTCAGATGGAACGTTGTAATGCCCAAGCTCATTGAGCACTCTATAACAATCCTCTCGGACGCCGGTCTCGGCATG<br>GCCATGTTCAGCTTAGGTGAGCTCCATTCTACAACTTGTTTCTAGCTCACAAATGTCAAAGCAGGACTTTTCATGGGATTGCAATCAAAGTTGTTAGCTTGC<br>GGTACTACCATGGCTATCGTAGTCATGATCATCCGTTTCGTCACCGGCCCAGCAATCATGTCCGCAACGTCAATAGCAGTCGGCCTCAGGAACGTCGAGCT<br>TAAGGCATCCATAGTTCAGGTAAACCTTCAATTAAAACGGTTAAATGGATTGAGTTCGTTAAACGTAACCCAGGCTGCACTACCACAAGGCATTGTCCCCT<br>TTGTCTTCGCCAAGGAGTACAACGTCCACCCTGACGTCCTCAGCACAGCGTAAGCATGTTTTATGTCCTGTGTATATAAATACGCTACCATTTTCAGGGTCA<br>TCTTCGGAATGTTGATCTCACTACCCATCGATCTTCTCTACTACGTACTGTTGGGC…

Development • Supplementary information
